# Supplementary material for: Benchmarking scRNA-seq copy number variation callers
Source: Nat Commun. 2025 Oct 2;16:8777. doi: 10.1038/s41467-025-62359-9 (PMC12491403; doi:10.1038/s41467-025-62359-9)
Supplement: Supplementary file 1 — Supplementary Information [file 41467_2025_62359_MOESM1_ESM.pdf]

Supplementary Information - Benchmarking scRNA-seq copy number variation callers

Katharina T. Schmid [1], Aikaterini Symeonidi [1,2], Dmytro Hlushchenko [1], Maria L. Richter [1], Andréa E. Tijhuis [3], Floris Foijer [3], Maria Colomé-Tatché [1,2]

1 Biomedical Center (BMC), Physiological Chemistry, Faculty of Medicine, LMU Munich, Planegg-Martinsried, Germany

2 Institute of Computational Biology, Computational Health Center, Helmholtz Zentrum München, German Research Center for Environmental Health, Neuherberg, Germany

3 European Research Institute for the Biology of Ageing, University of Groningen, University Medical Center Groningen, Groningen, The Netherlands

Supplementary Tables

Expression threshold	Pearson correlation	Maximal F1 score	Partial AUC (gain)	Partial AUC (loss)
0.1	0.22	0.57	0.63	0.59
4.5	0.64	0.68	0.83	0.86

Supplementary Table 1. Performance differences for CaSpER depending on the chosen expression threshold for the SNU601 cell line. The expression filtering is directly defined within CaSpER (function CreateCasperObjec(), parameter expr.threshold). All genes whose mean expression is smaller than the threshold are removed from the analysis. Recommended cutoff based on the 10X tutorial of CaSpER is 0.1, default parameter for bulk and plate-based single cell data is 4.5.

Supplementary Figures

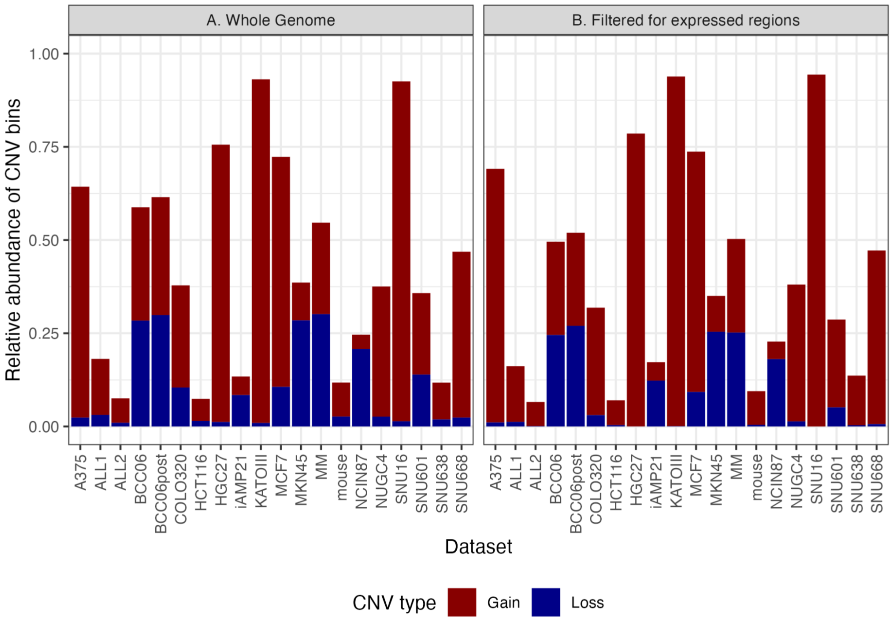

Supplementary Figure 1. CNV distribution across datasets. The CNV type is assigned based on the genomic ground truth ((sc)WGS or WES). The distribution for all n=20 cancer datasets is shown both A. for the CNVs in the whole genome, i.e. the whole area for which we have genomic information available for this dataset, and B. for the CNVS in the area of the genome which is covered by the scRNA-seq CNV callers. Source data are provided as a Source Data file.

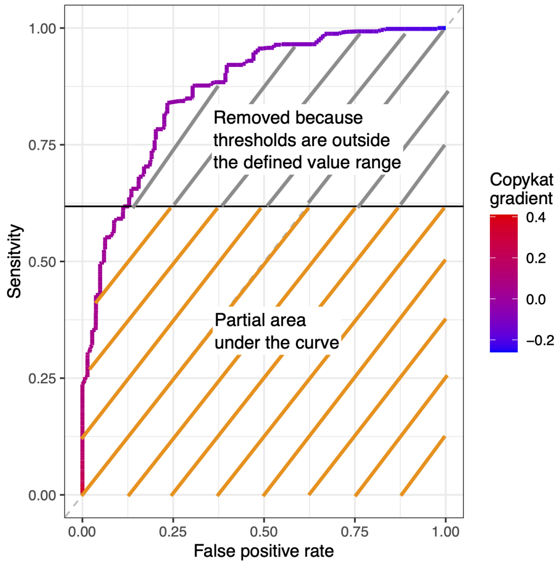

Supplementary Figure 2. Illustration of the partial AUC scores. Shown for the ROC curve for CopyKat, for identified gains applied to the MCF7 dataset. The area under the curve (AUC) is only calculated for thresholds which are inside the allowed value range for the CNV type and method. For gains, the allowed thresholds are above 0 (for losses, the allowed thresholds would be below 0). The vertical line visualizes the sensitivity when using 0 as threshold. Source data are provided as a Source Data file.

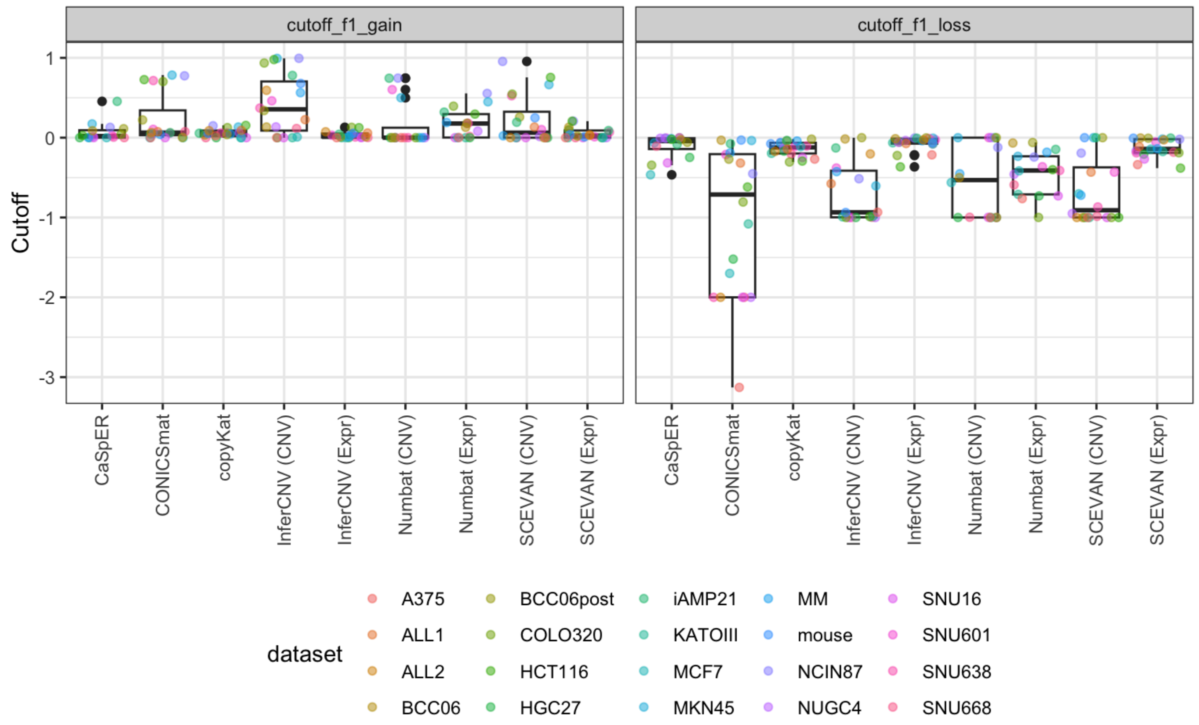

Supplementary Figure 3. Optimal gain and loss thresholds according to the multi-class F1 evaluation. The baseline value of each method is set to 0 and the analysis performed for n=20 cancer datasets. The boxplots show medians (center lines), first and third quartiles (lower and upper box limits, respectively), 1.5-fold interquartile ranges (whisker extents) and outliers (black dots). Source data are provided as a Source Data file.

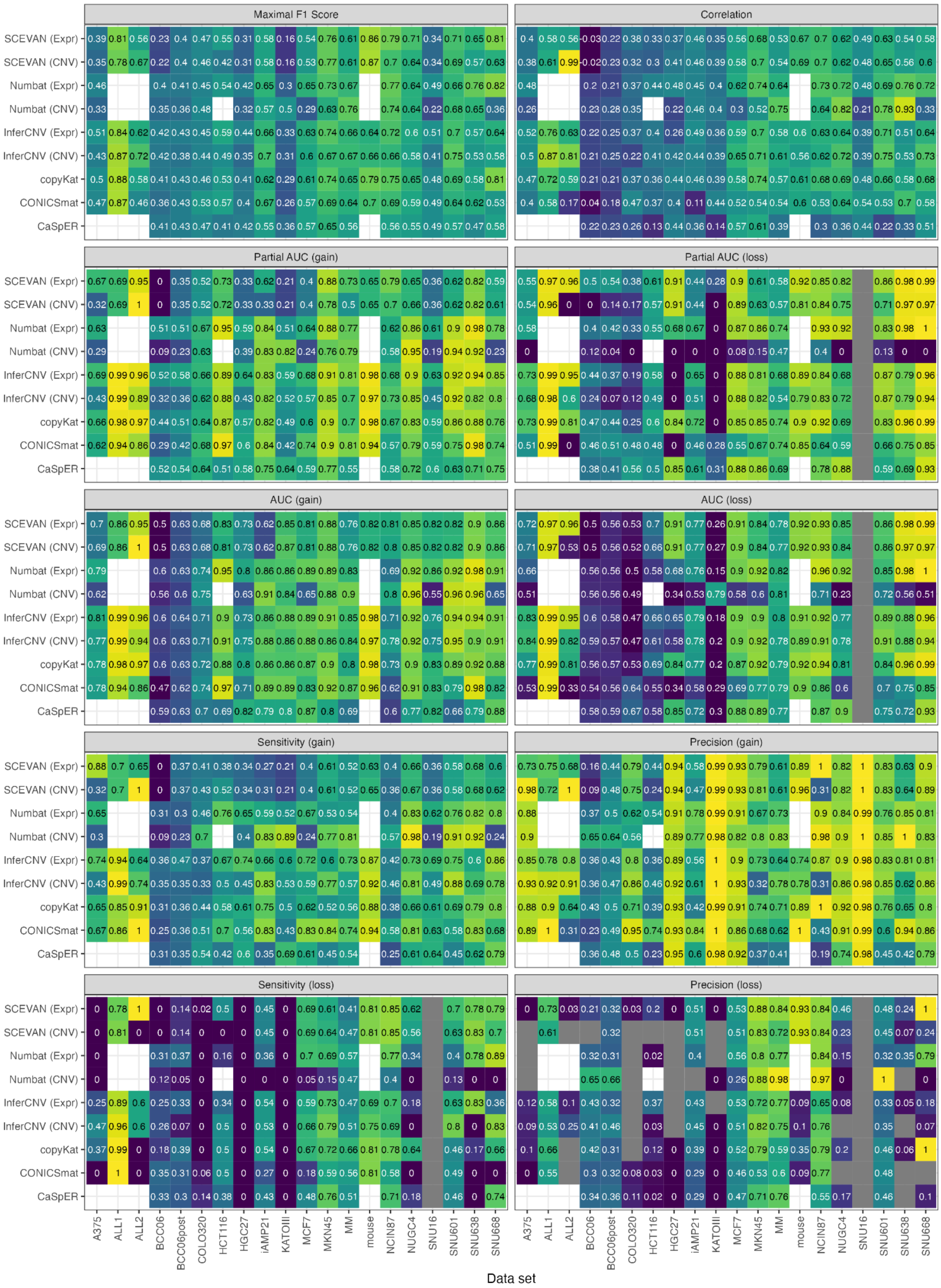

Supplementary Figure 4. Additional evaluation metrics for all datasets. The AUC values were calculated separately for gains vs all and loss vs all, same for the sensitivity and precision values. Cutoffs for gains and losses were chosen based on maximal F1 scores. Due to lack of genomic information, the ALL1, ALL2 and mouse data were not run with CaSpER and Numbat. For the dataset A375, CaSpER identified no CNVs, for HCT116, Numbat (CNV) identified no CNVs. Source data are provided as a Source Data file.

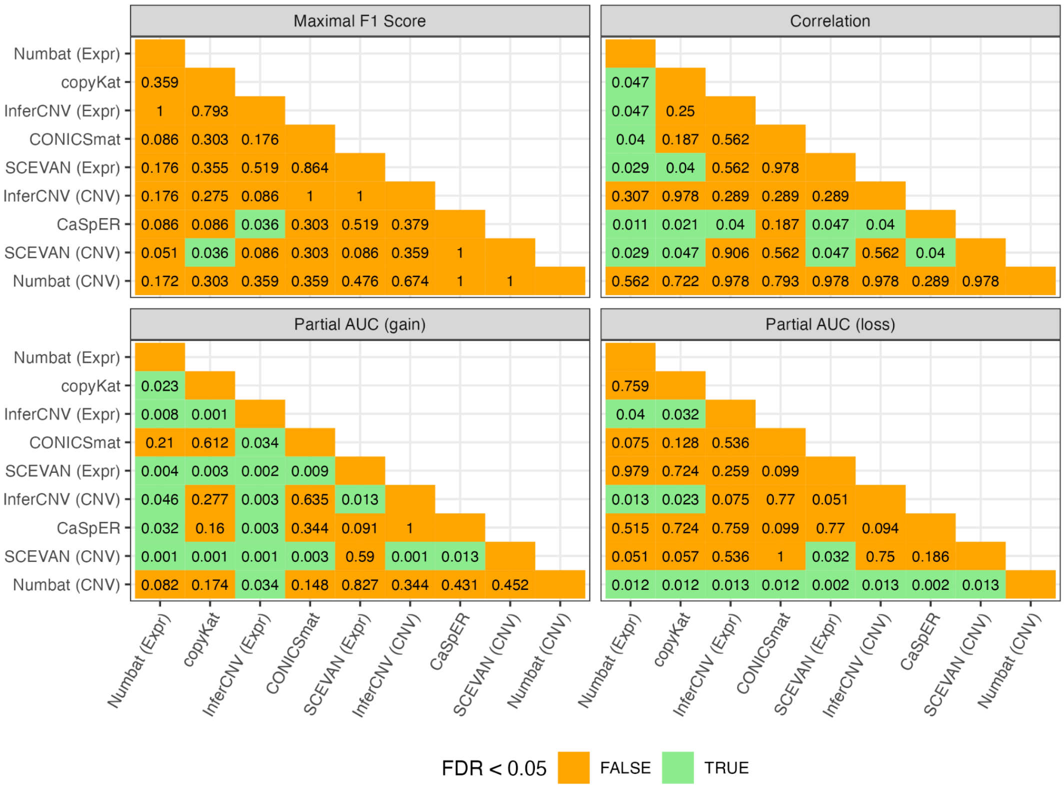

Supplementary Figure 5. Method comparison across datasets. FDR-corrected p-values from Wilcoxon signed rank tests (two-sided) to compare the method's performance across droplet-based human datasets (n=15); pairwise for each combination of methods. Source data are provided as a Source Data file.

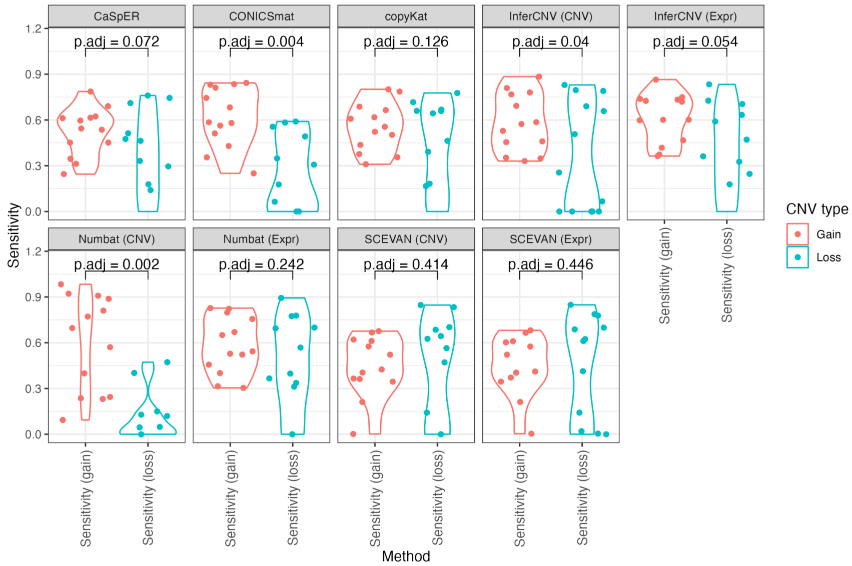

Supplementary Figure 6. Comparison of sensitivity for gains vs losses for all methods. Each dot represents one datasets from the droplet-based human cancer datasets (n=15). "p.adj" represents the FDR-corrected p-values from Wilcoxon signed rank tests (one-sided). Source data are provided as a Source Data file.

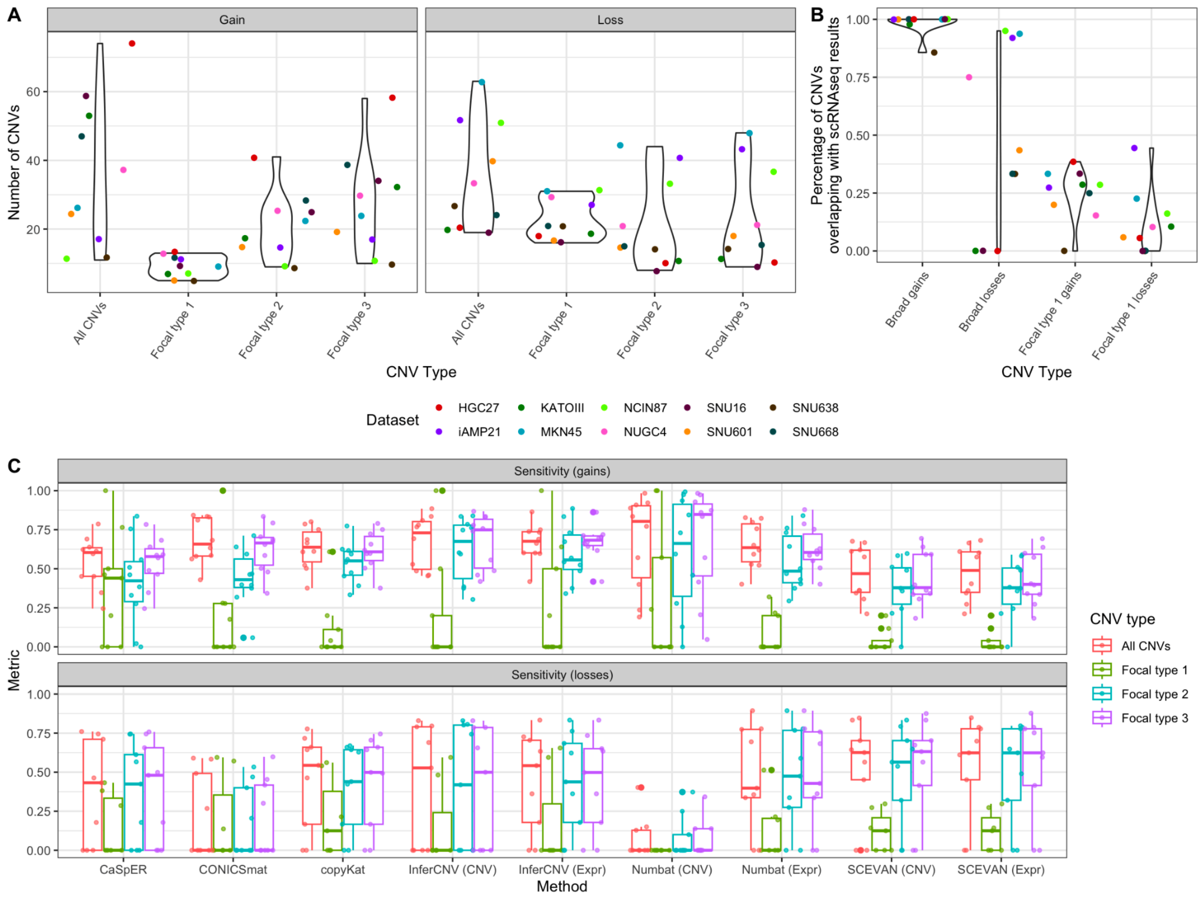

Supplementary Figure 7. Method performance on focal CNVs. Considering all droplet-based datasets with scWGS ground truth (n=10). A. Number of total and focal CNVs identified from scWGS based on three different focal definitions: focal type 1 (size < 3Mb), focal type 2 (size < 50% of a chromosome arm) and focal type 3 (size < 95% of a chromosome arm). B. Percentage of CNVs that overlap the analysed regions of the scRNA-seq CNV callers for focal type 1 CNVs and broad CNVs (all CNVs > 3Mb). C. Sensitivity in identifying gains and losses for each of the focal types. The boxplots show medians (center lines), first and third quartiles (lower and upper box limits, respectively), 1.5-fold interquartile ranges (whisker extents) and outliers (black dots). Source data are provided as a Source Data file.

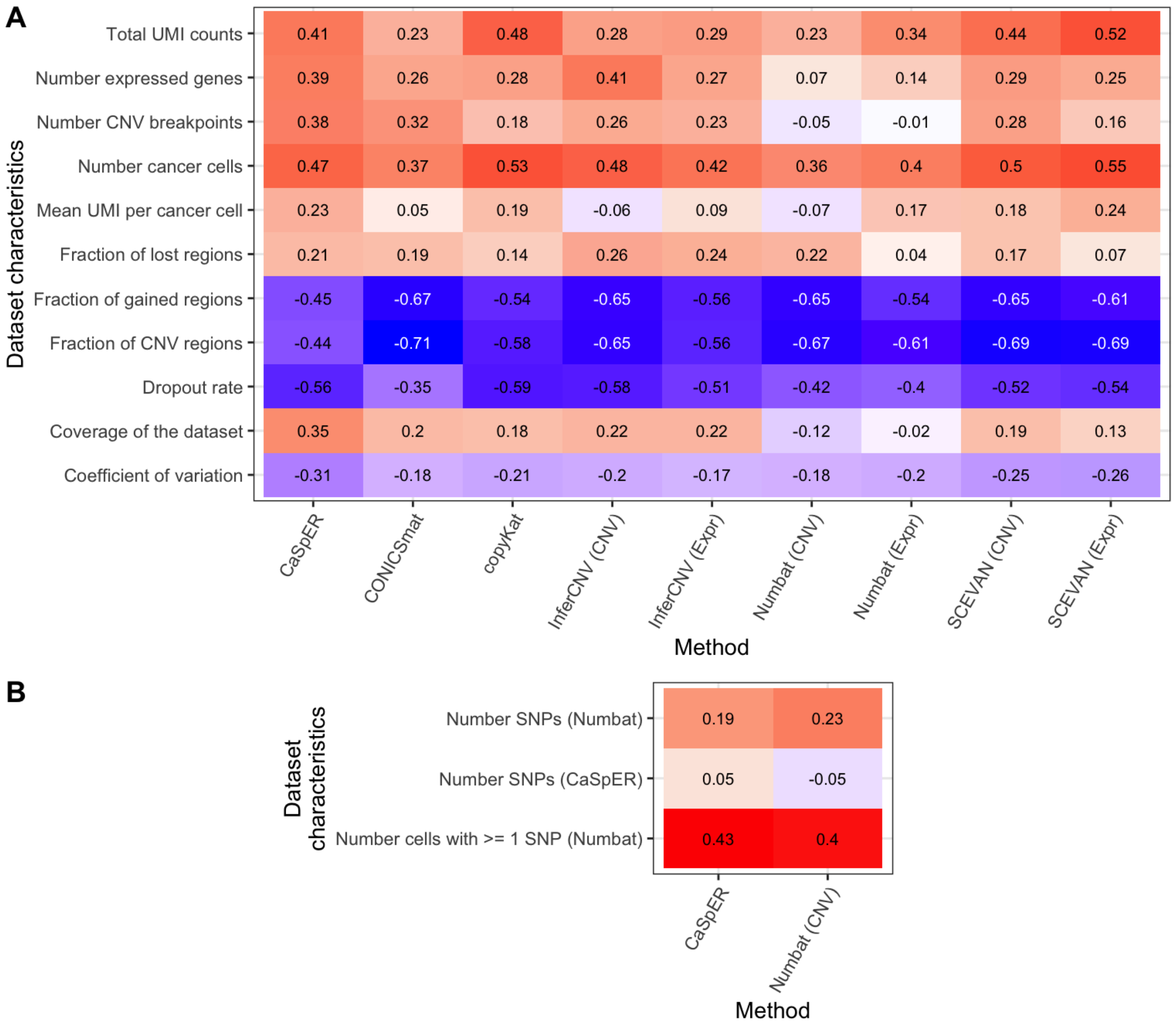

	CaSpER	CONICSmat	copyKat	InferCNV (CNV)	InferCNV (Expr)	Numbat (CNV)	Numbat (Expr)	SCEVAN (CNV)	SCEVAN (Expr)
Total UMI counts	0.41	0.23	0.48	0.28	0.29	0.23	0.34	0.44	0.52
Number expressed genes	0.39	0.26	0.28	0.41	0.27	0.07	0.14	0.29	0.25
Number CNV breakpoints	0.38	0.32	0.18	0.26	0.23	-0.05	-0.01	0.28	0.16
Number cancer cells	0.47	0.37	0.53	0.48	0.42	0.36	0.4	0.5	0.55
Mean UMI per cancer cell	0.23	0.05	0.19	-0.06	0.09	-0.07	0.17	0.18	0.24
Fraction of lost regions	0.21	0.19	0.14	0.26	0.24	0.22	0.04	0.17	0.07
Fraction of gained regions	-0.45	-0.67	-0.54	-0.65	-0.56	-0.65	-0.54	-0.65	-0.61
Fraction of CNV regions	-0.44	-0.71	-0.58	-0.65	-0.56	-0.67	-0.61	-0.69	-0.69
Dropout rate	-0.56	-0.35	-0.59	-0.58	-0.51	-0.42	-0.4	-0.52	-0.54
Coverage of the dataset	0.35	0.2	0.18	0.22	0.22	-0.12	-0.02	0.19	0.13
Coefficient of variation	-0.31	-0.18	-0.21	-0.2	-0.17	-0.18	-0.2	-0.25	-0.26

	CaSpER	Numbat (CNV)
Number SNPs (Numbat)	0.19	0.23
Number SNPs (CaSpER)	0.05	-0.05
Number cells with >= 1 SNP (Numbat)	0.43	0.4

Supplementary Figure 8. Correlation of different dataset characteristics with the prediction performance (maximal F1 score). The analysis considers all human droplet-based datasets (n=15) and is subdivided into general dataset characteristics (A) and dataset characteristics related to SNP calling (B), which are only affecting CaSpER and Numbat (CNV). Detailed description of all dataset characteristics found in Methods section. Source data are provided as a Source Data file.

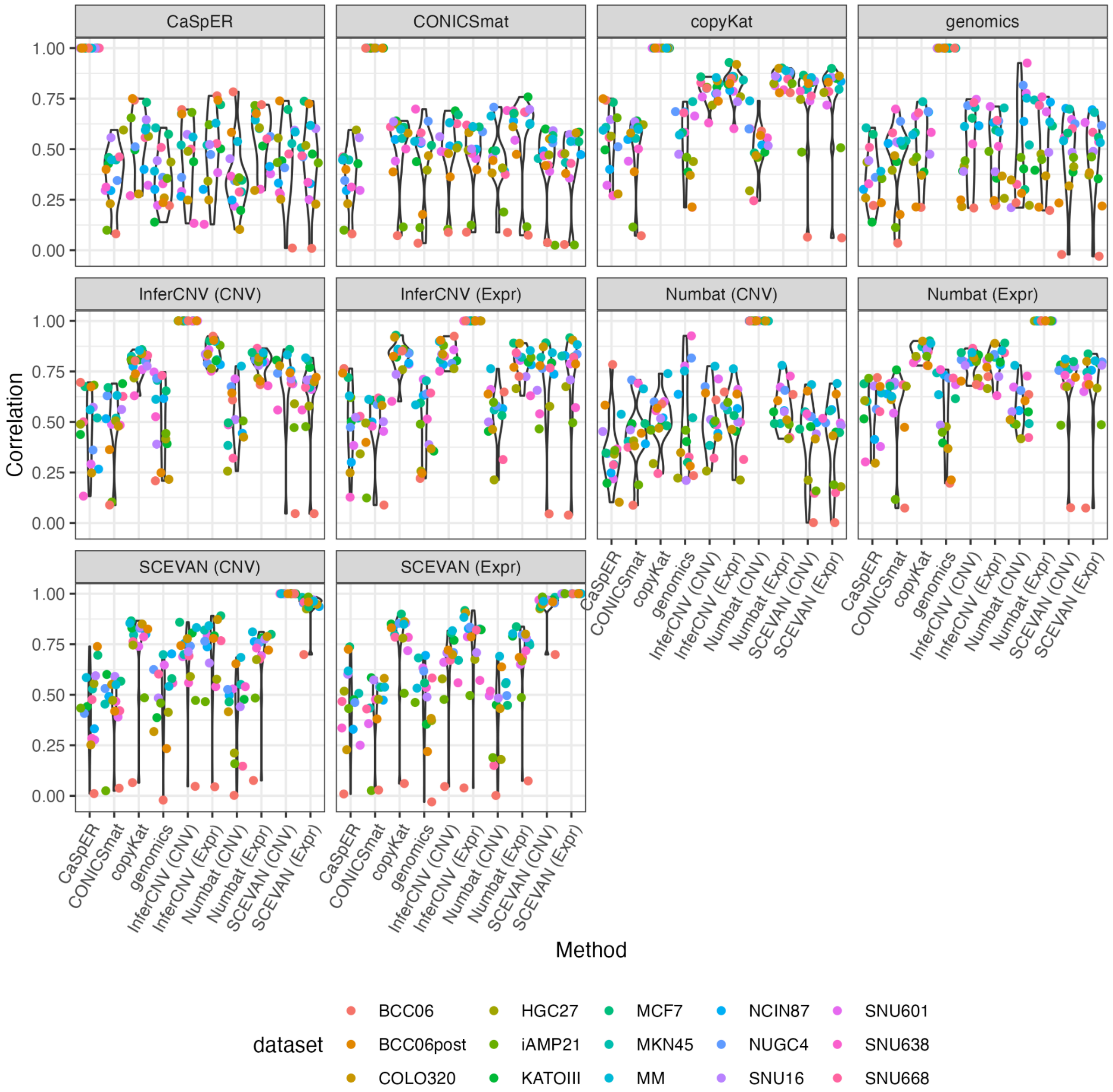

Supplementary Figure 9. Comparison of CNV predictions across scRNA-seq callers. We quantified similarities between methods across all human droplet-based cancer datasets (n=15) using Pearson correlation. Source data are provided as a Source Data file.

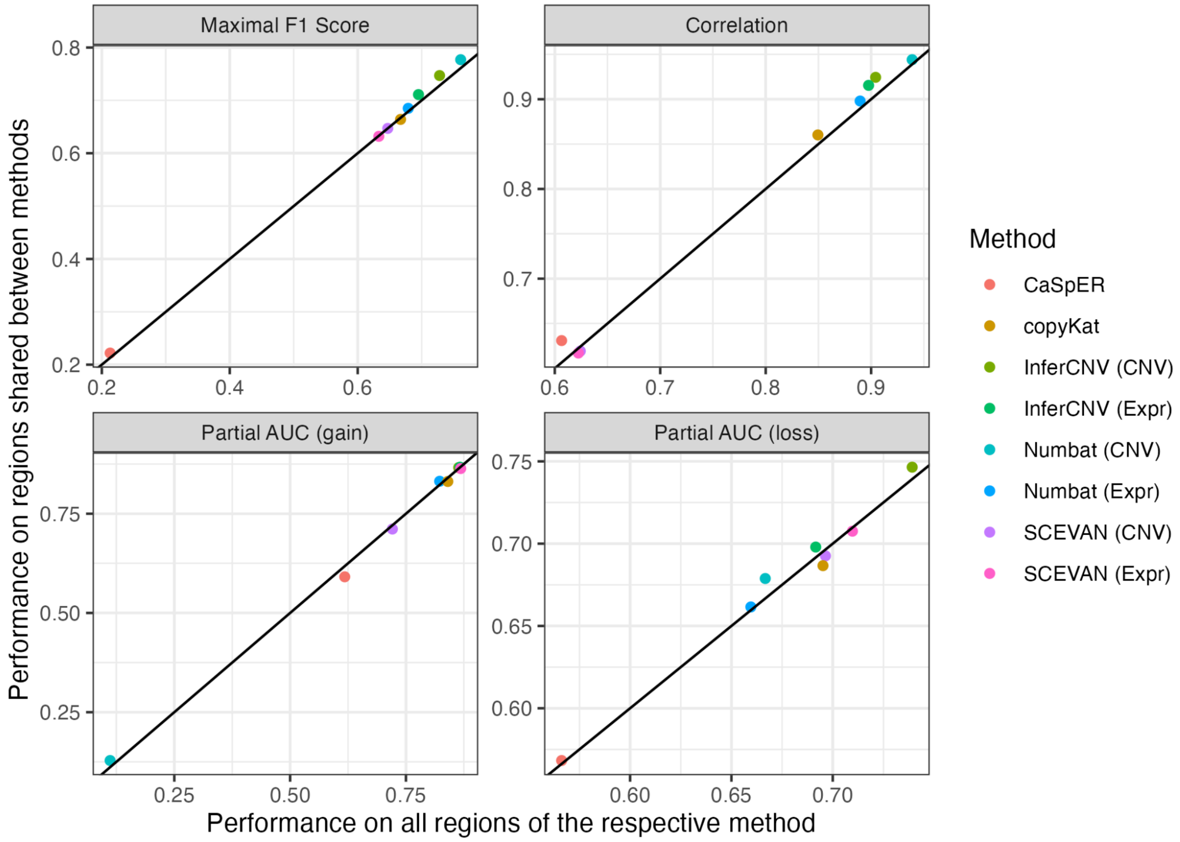

Supplementary Figure 10. Performance differences depending on shared genomic regions. We repeated the performance evaluation, when looking into regions that are annotated by all methods (y axis) vs. all regions that are annotated by the respective method (x axis). Source data are provided as a Source Data file.

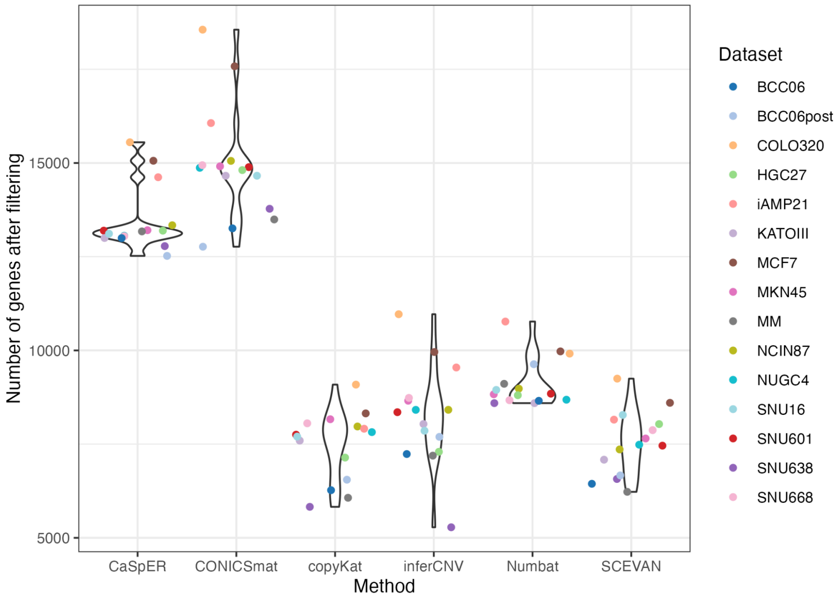

Supplementary Figure 11. Number of genes retained after filtering. For each method per dataset for all human droplet-based cancer datasets (n=15). Source data are provided as a Source Data file.

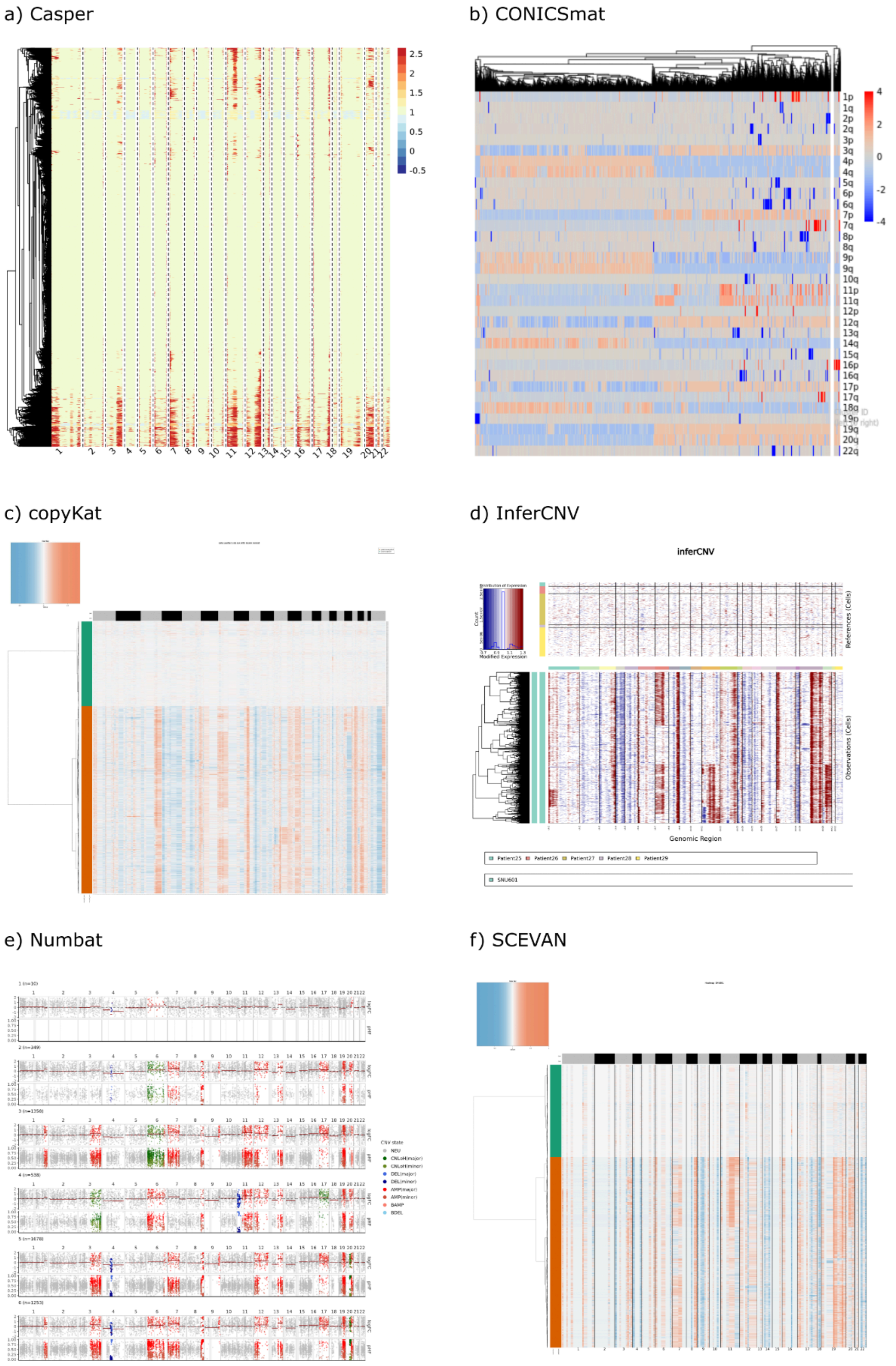

Supplementary Figure 12. Example result plots from each method for the SNU601 dataset. One representative plot was chosen per method.

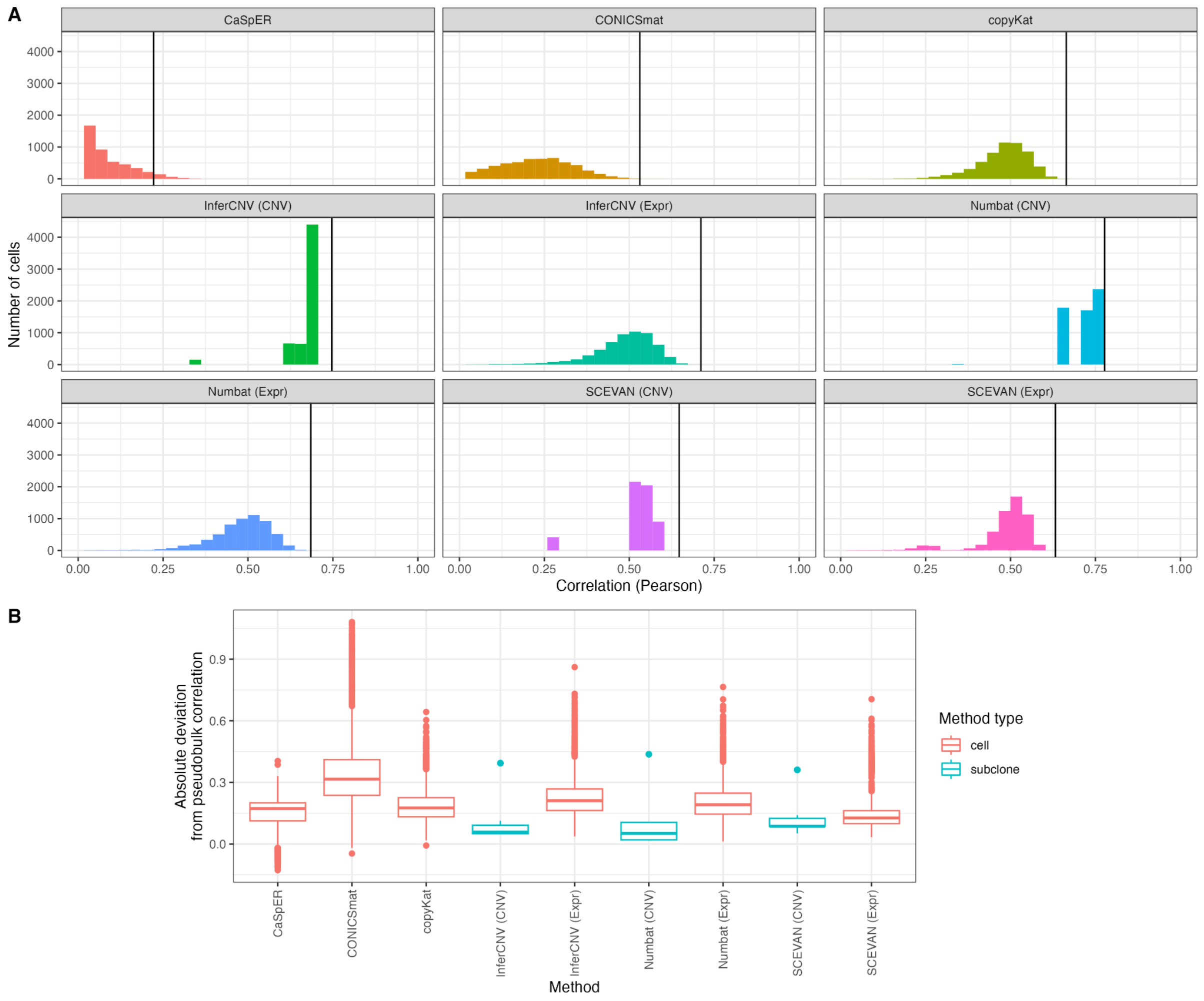

Supplementary Figure 13. Per cell prediction performance for all methods compared to the scWGS ground-truth for the SNU601 cell line (n=5880 cells). **(A)** Histogram of per cell correlation with the ground truth, vertical lines show the performance of the pseudobulk aggregated prediction. For the methods which report subclonal CNV profiles instead of per cell CNV, the subclonal results were replicated by the number of cells in the respective subclone. Correlation shown only for the value range 0-1 for better visibility, removing negative outliers. **(B)** Deviation of per cell correlation values from the pseudobulk correlation value, color-coded by whether the method reports per cell results or subclonal results. The boxplots show medians (center lines), first and third quartiles (lower and upper box limits, respectively), 1.5-fold interquartile ranges (whisker extents) and outliers (black dots). Source data are provided as a Source Data file.

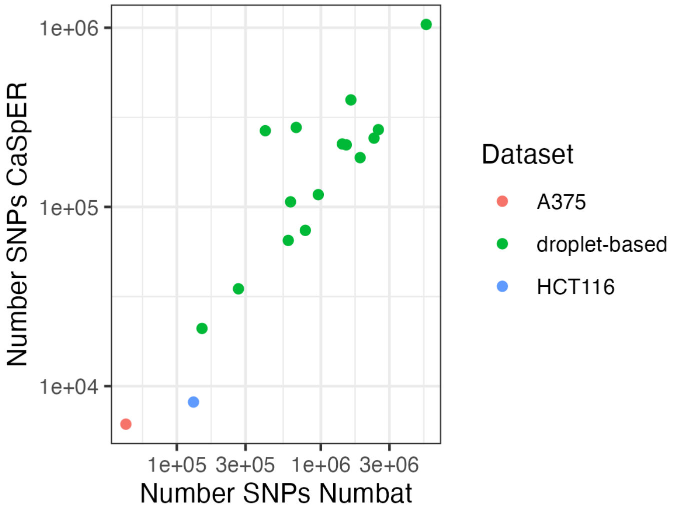

Supplementary Figure 14. Number of SNPs identified with CaSpER and Numbat depending on the single cell technology. Comparison of the number of SNPs (axes in log scale) for the two DNTR-seq based methods A375 and HCT116 with the human droplet-based datasets (n=15) analyzed in this benchmarking. Source data are provided as a Source Data file.

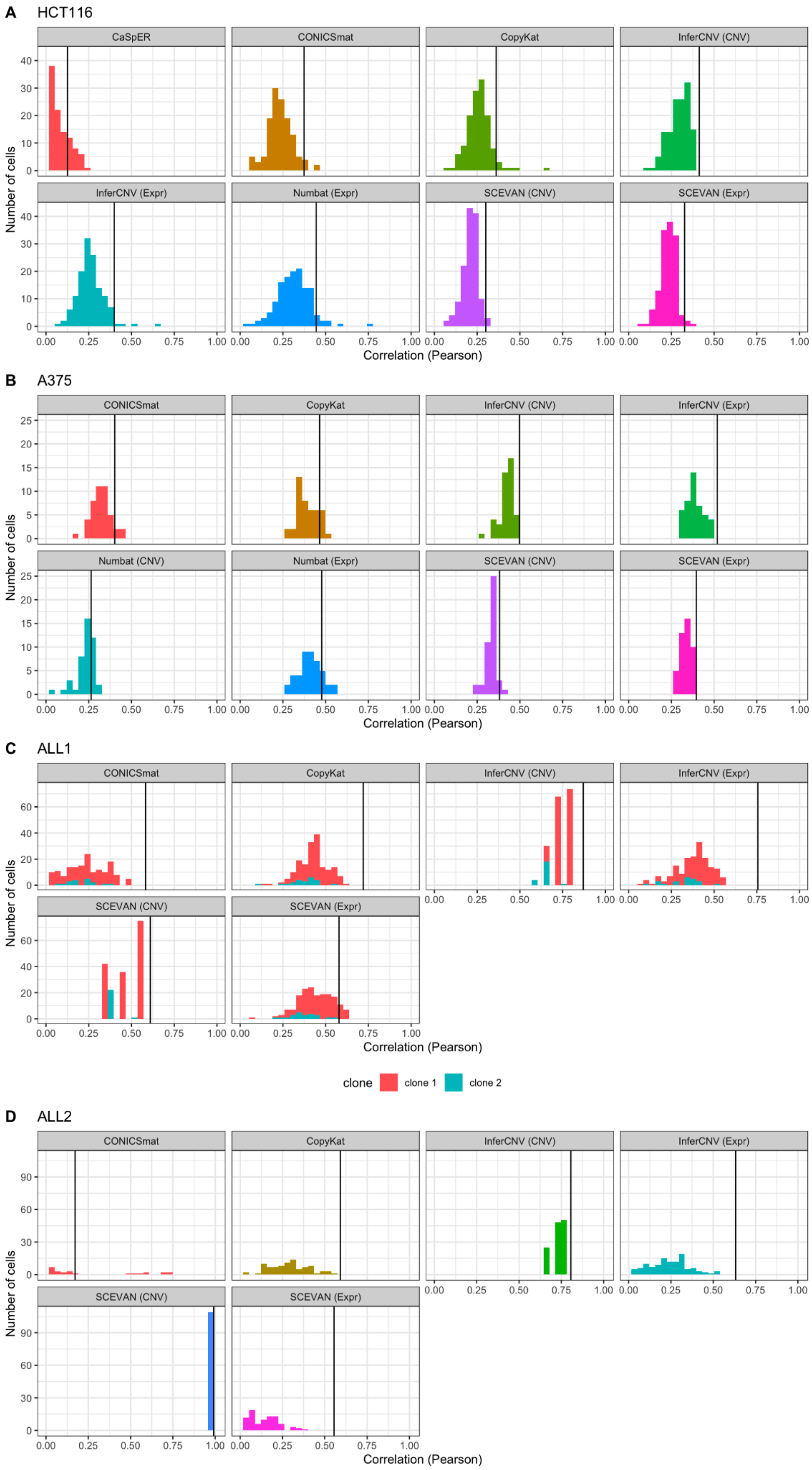

Supplementary Figure 15. Legend on the next page

Supplementary Figure 15. Per cell performance results (Pearson correlation) with matched cells for the four DNTR-seq datasets. HCT116 (n=134 cells) (**A**), A375 (n=44 cells) (**B**), ALL1 (n=177 cells) splitted after the two subclones (**C**) and ALL2 (n=123 cells) (**D**). The vertical black line visualizes the correlation to the pseudobulk. For the HCT116 (**A**), Numbat (CNV) is missing here, as no CNVs were found for this dataset, so no correlation could be calculated. For the same reason, CaSpER is missing for the A375 (**B**) (no CNVs found with this method). For ALL1 and ALL2 (**C+D**), only the expression-based methods were tested. Source data are provided as a Source Data file.

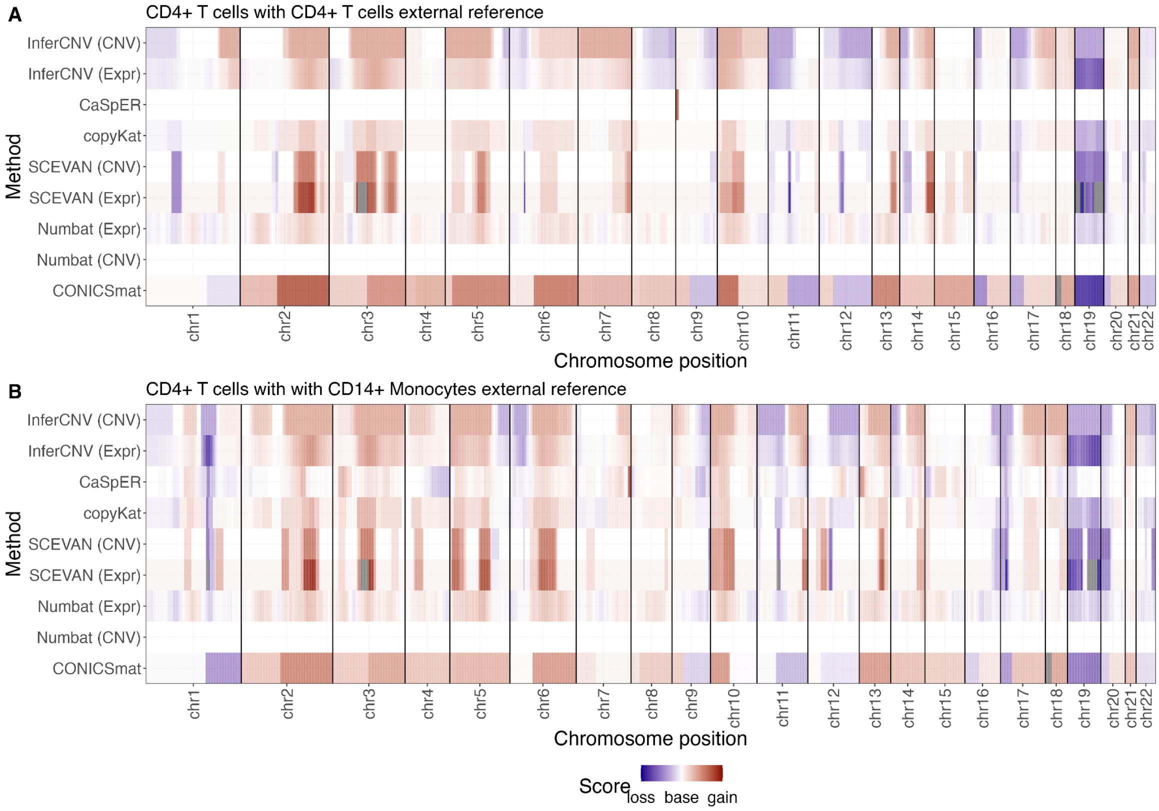

Supplementary Figure 16. CNV calling on euploid datasets with an external reference. Karyograms of CNVs in CD4+ T cells when using either CD4+ T cells (A) or CD14+ Monocytes (B) from an external dataset as reference. Source data are provided as a Source Data file.

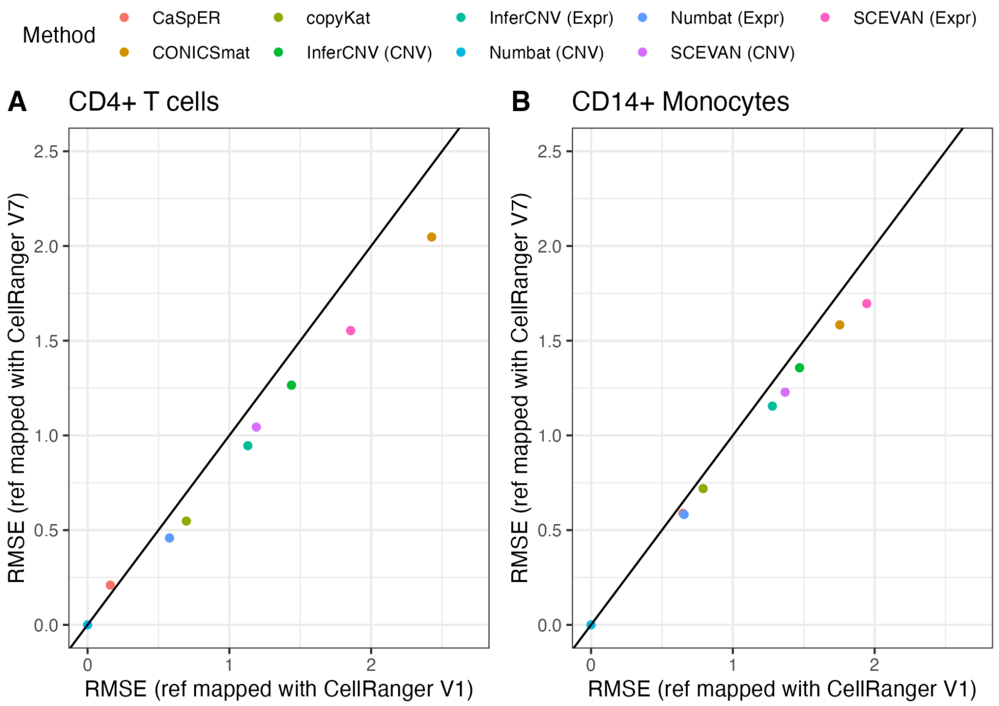

Supplementary Figure 17. Performance difference in euploid datasets depending on the mapping tool. Comparison of root mean square errors (RMSE) for identifying CNVs in a diploid dataset of CD4+ T cells when using two different mapper versions for the reference dataset: the reference contained CD4+ T cells (A) and CD14+ Monocytes (B), respectively, from a different study, once mapped with CellRanger version 1 and once with CellRanger version 7. Source data are provided as a Source Data file.

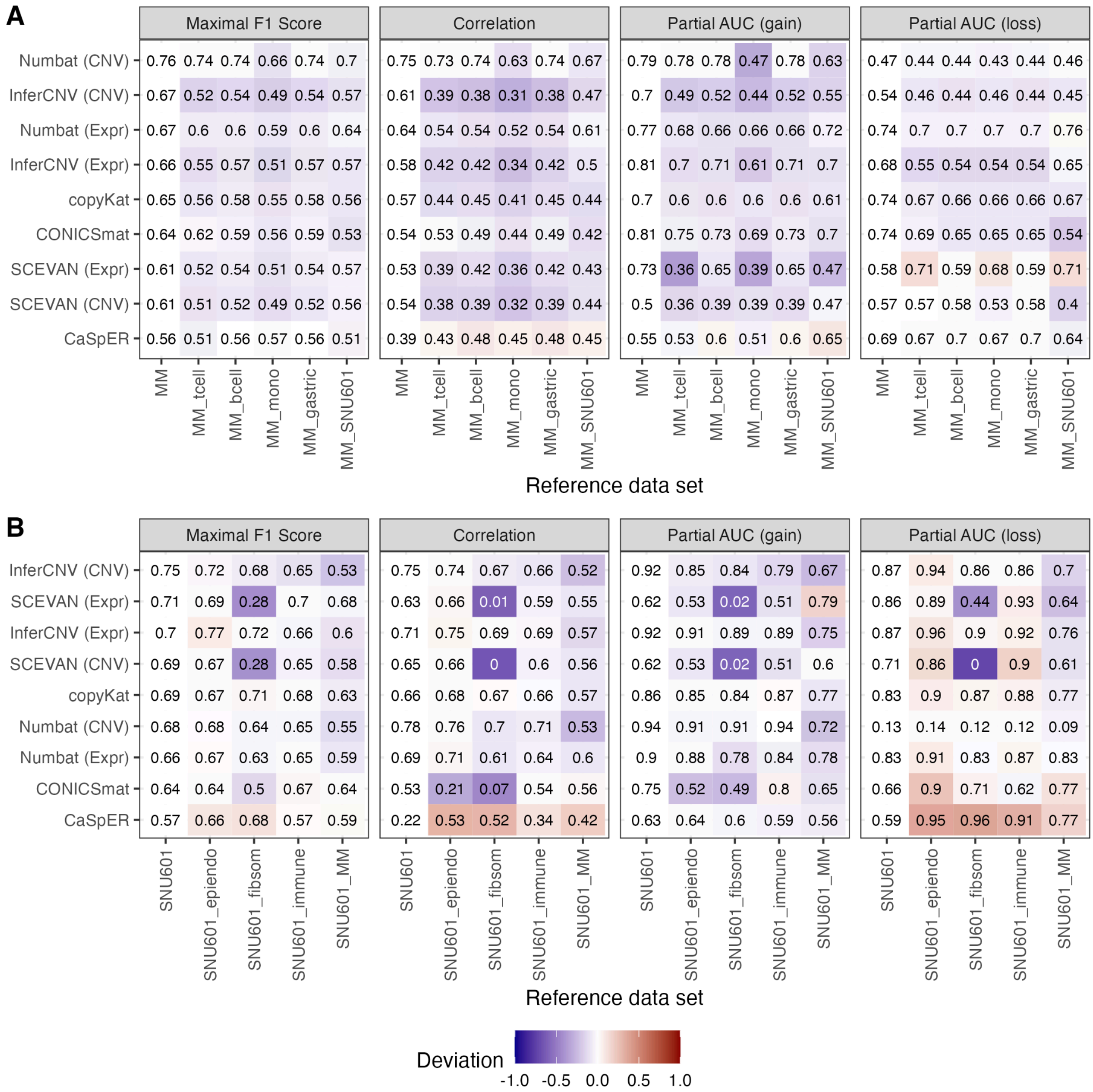

Supplementary Figure 18. Performance differences in aneuploid datasets depending on the reference. Performance for predicting CNVs in the MM dataset (A) and the SNU601 dataset (B), when using different reference datasets. The color gradient in the plot shows the deviation of each metric from the first column of the respective panel, in which the reference dataset initially chosen by us in the benchmarking is displayed. We selected this initial reference dataset, as it was biologically the closest cell type for the normalization. Source data are provided as a Source Data file.

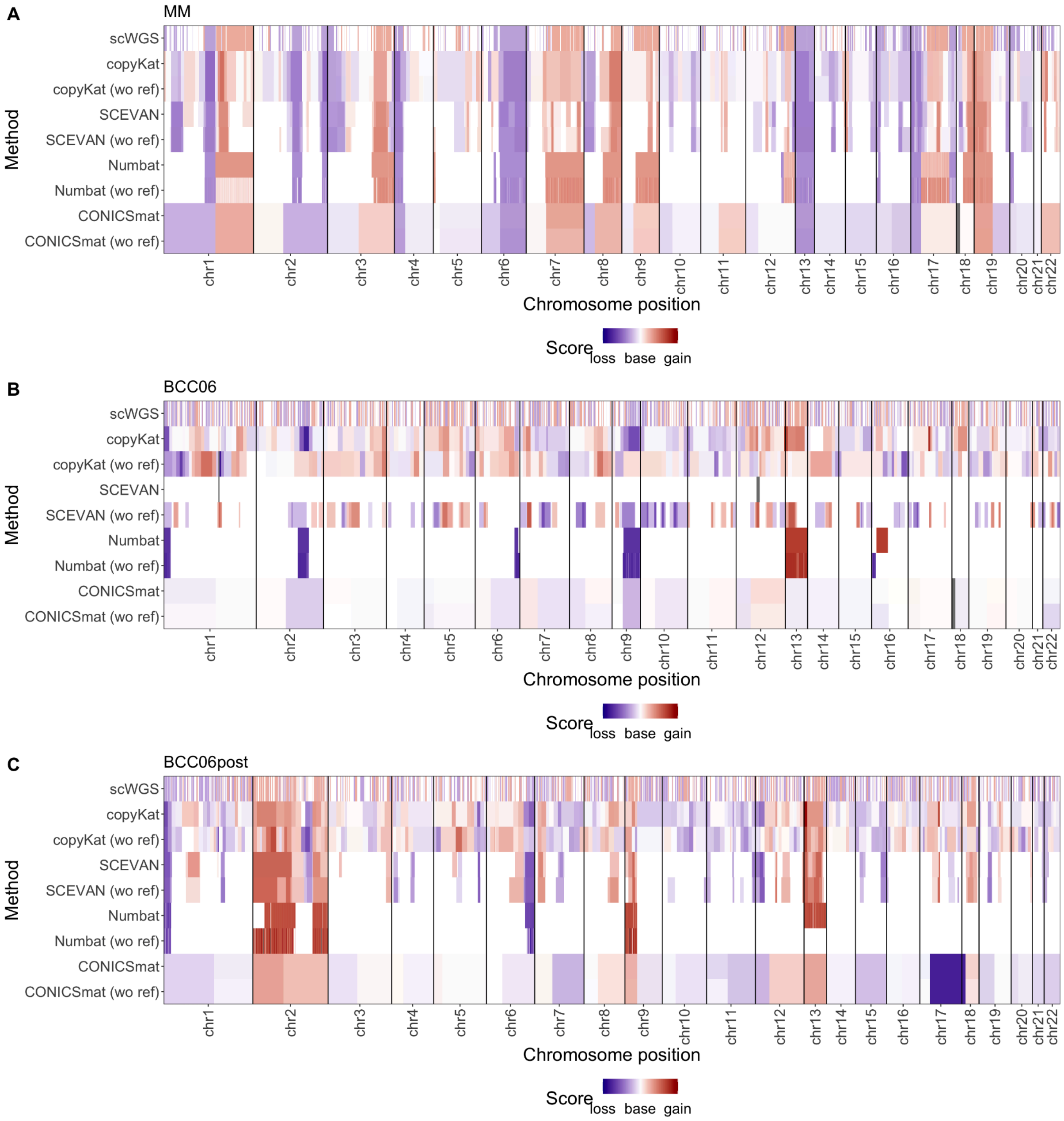

Supplementary Figure 19. Performance differences between manual and automatic reference. Karyograms showing the difference between using a manual reference and an automatic one for the datasets MM (A), BCC06 (B) and BCC06post (C). Source data are provided as a Source Data file.

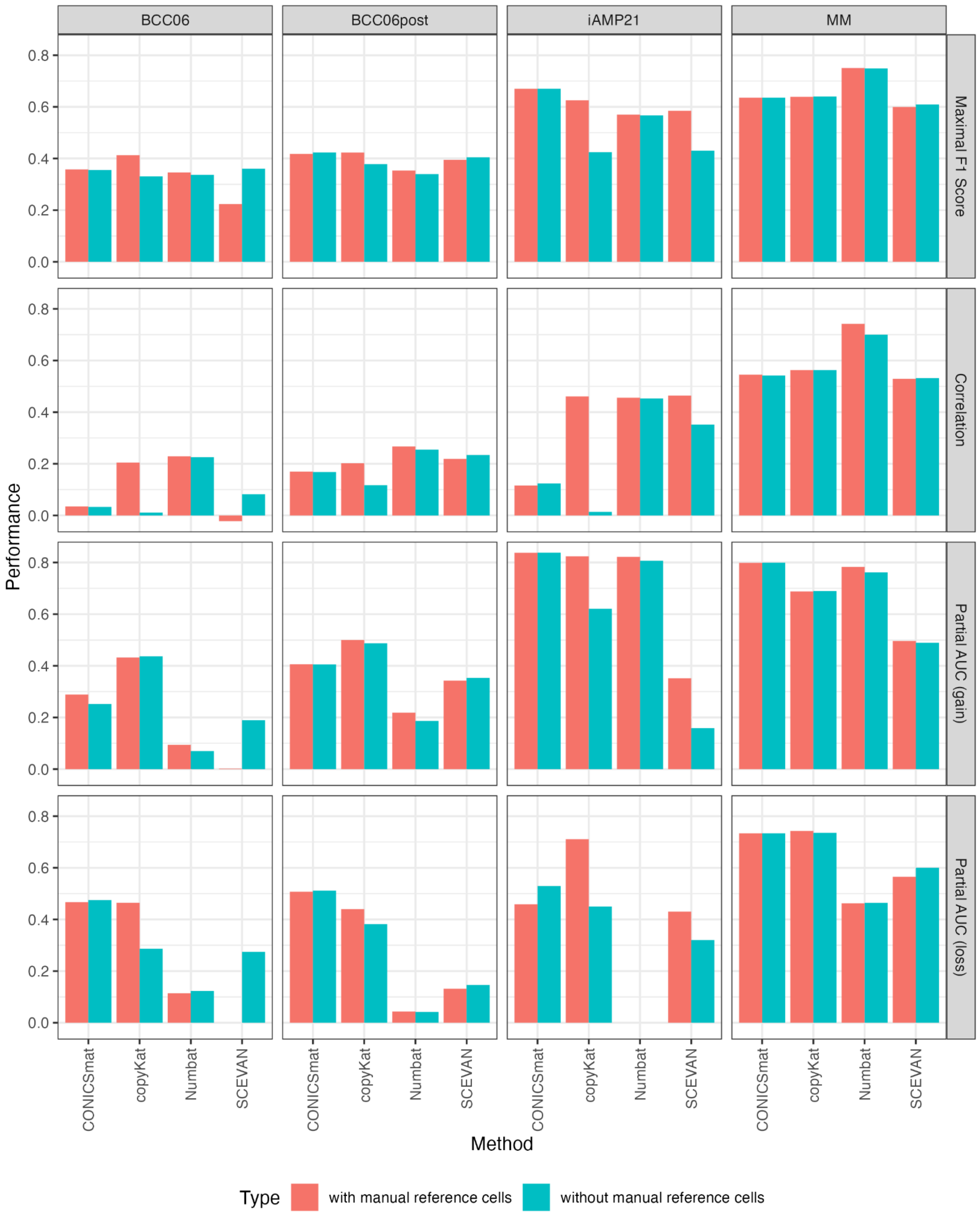

Supplementary Figure 20. Performance differences between manual and automatic reference. Comparing CNV prediction performance when running copyKat, SCEVAN, Numbat and CONICSmat with and without manual reference cells. For the MM and the BCC06post samples, there is a high concordance of cell type annotations between the scenarios “with manual reference cells” and “without manual reference cells”, which is very likely the explanation of why the performance between the two is not differing much. Source data are provided as a Source Data file.

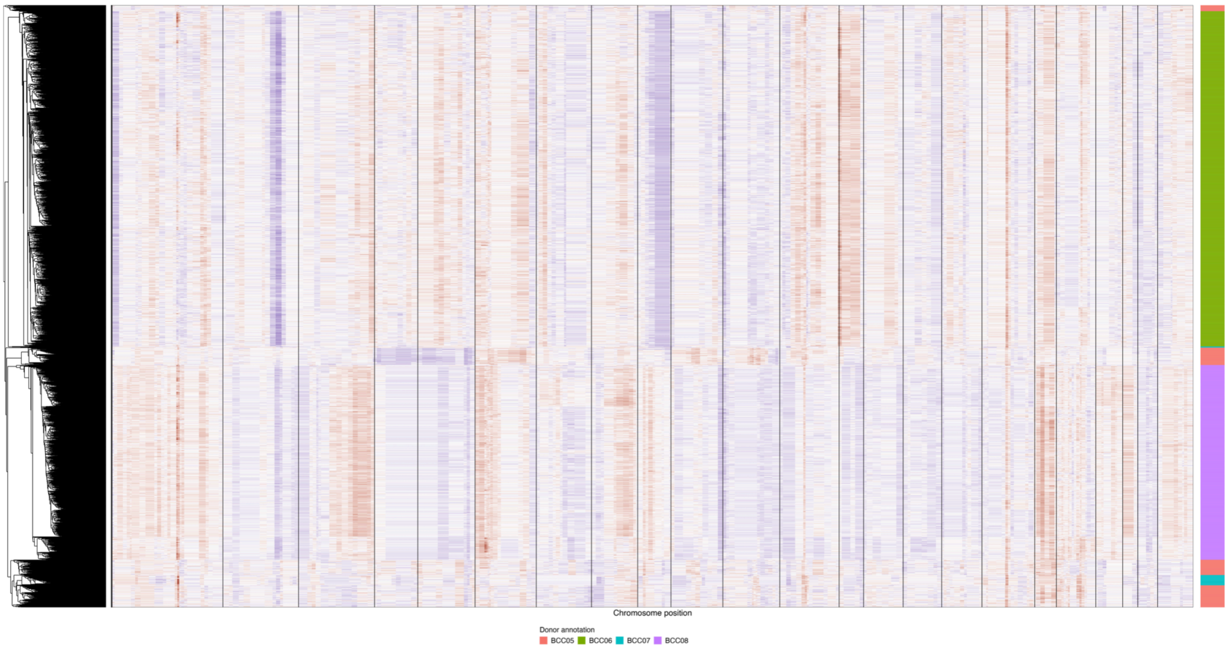

Supplementary Figure 21. Per cell CNV profile of the four BCC donors. Estimated separately with copyKat and then clustered together afterwards.

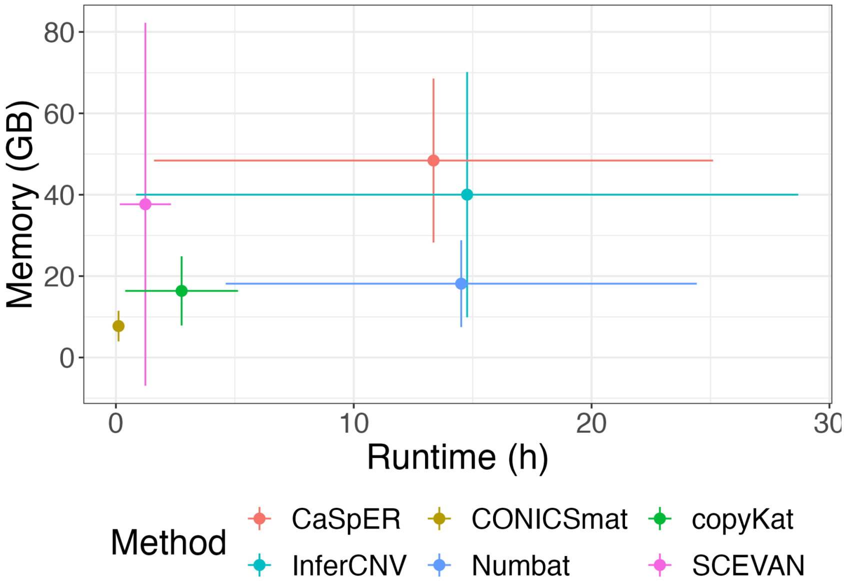

Supplementary Figure 22. Runtime and memory consumption of each method. The results are visualized across all cancer datasets (n=20), representing the mean consumption with the dot and minimum and maximum values with the horizontal and vertical lines. Source data are provided as a Source Data file.
