# Supplementary material for: Benchmarking scRNA-seq copy number variation callers
Source: Nat Commun. 2025 Oct 2;16:8777. doi: 10.1038/s41467-025-62359-9 (PMC12491403; doi:10.1038/s41467-025-62359-9)
Supplement: Supplementary file 2 — Description of Additional Supplementary Information [file 41467_2025_62359_MOESM2_ESM.docx]

**Description of Additional Supplementary Files**

File Name: Supplementary Data 1

Description: All datasets evaluated in the benchmarking.

File Name: Supplementary Data 2

Description: Karyograms of all tested human cancer datasets, measured with droplet-based scRNAseq.

File Name: Supplementary Data 3

Description: Karyograms of the HCT116 dataset per cell, for the scWGS and scRNA-seq results, split by method.

File Name: Supplementary Data 4

Description: Karyograms of the A375 dataset per cell, for the scWGS and scRNA-seq results, split by method.

File Name: Supplementary Data 5

Description: Karyograms of the ALL1 dataset per cell, for the scWGS and scRNA-seq results, split by method.

File Name: Supplementary Data 6

Description: Karyograms of the ALL2 dataset per cell, for the scWGS and scRNA-seq results, split by method.
