# Supplementary material for: Benchmarking scRNA-seq copy number variation callers
Source: Nat Commun. 2025 Oct 2;16:8777. doi: 10.1038/s41467-025-62359-9 (PMC12491403; doi:10.1038/s41467-025-62359-9)
Supplement: Supplementary file 8 — Supplementary Dataset 6 [file 41467_2025_62359_MOESM8_ESM.pdf]

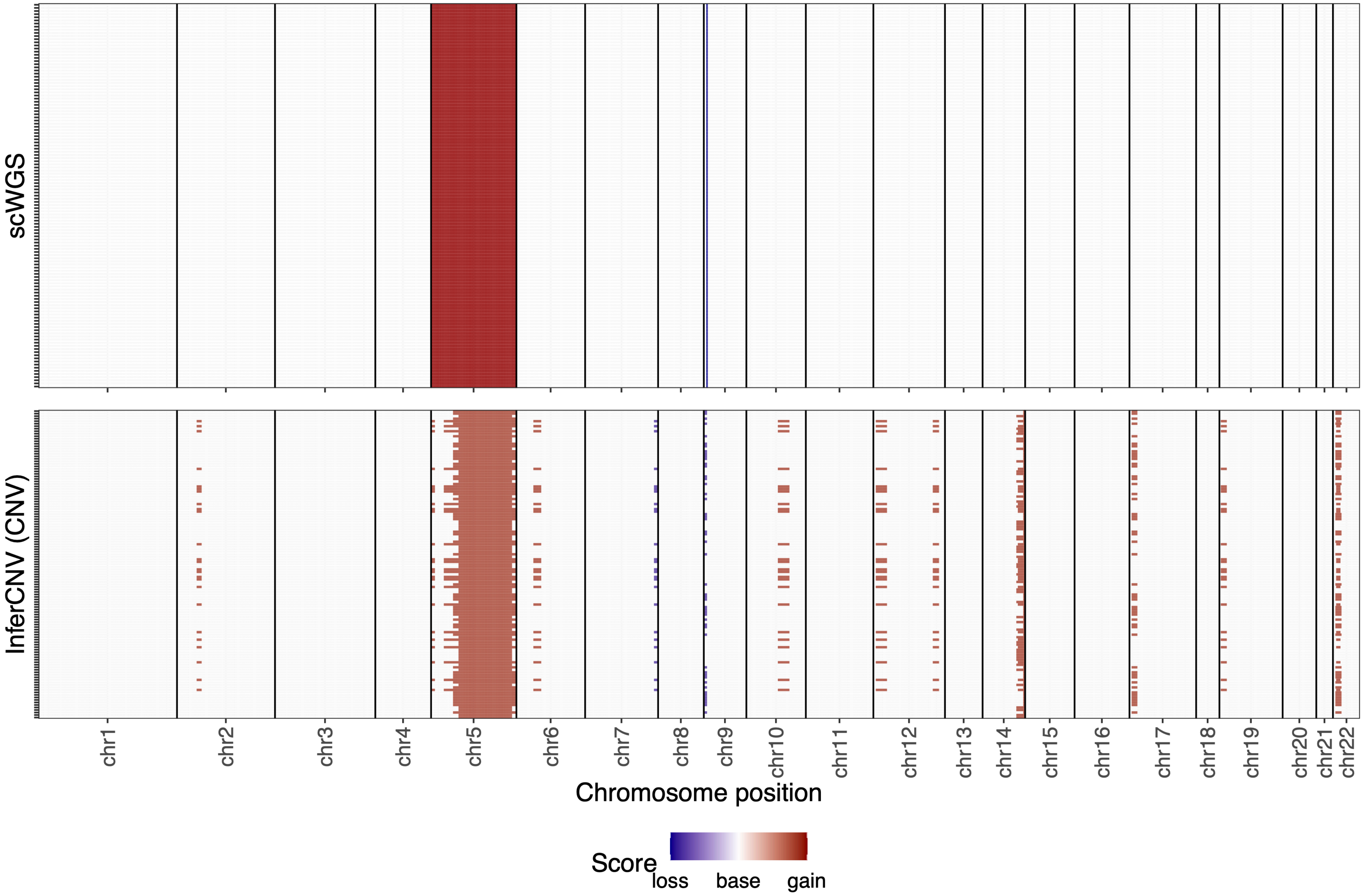
scWGS
InferCNV (CNV)
chr1
chr2
chr3
chr4
chr5
chr6
chr7
chr8
chr9
chr10
chr11
chr12
chr13
chr14
chr15
chr16
chr17
chr18
chr19
chr20
chr21
chr22
Chromosome position
Score
loss
base
gain

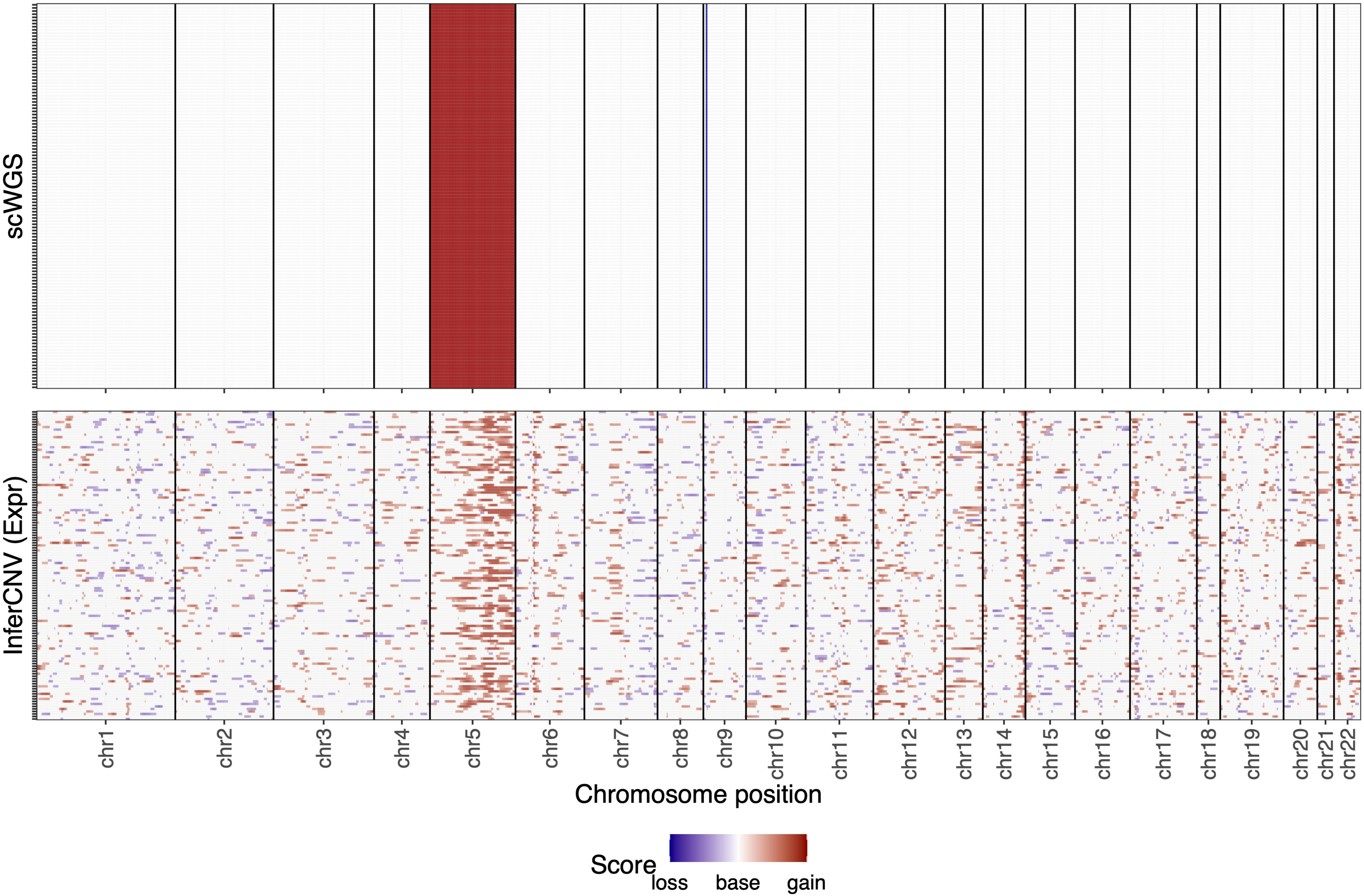
scWGS
InferCNV (Expr)
chr1
chr2
chr3
chr4
chr5
chr6
chr7
chr8
chr9
chr10
chr11
chr12
chr13
chr14
chr15
chr16
chr17
chr18
chr19
chr20
chr21
chr22
Chromosome position
Score
loss
base
gain

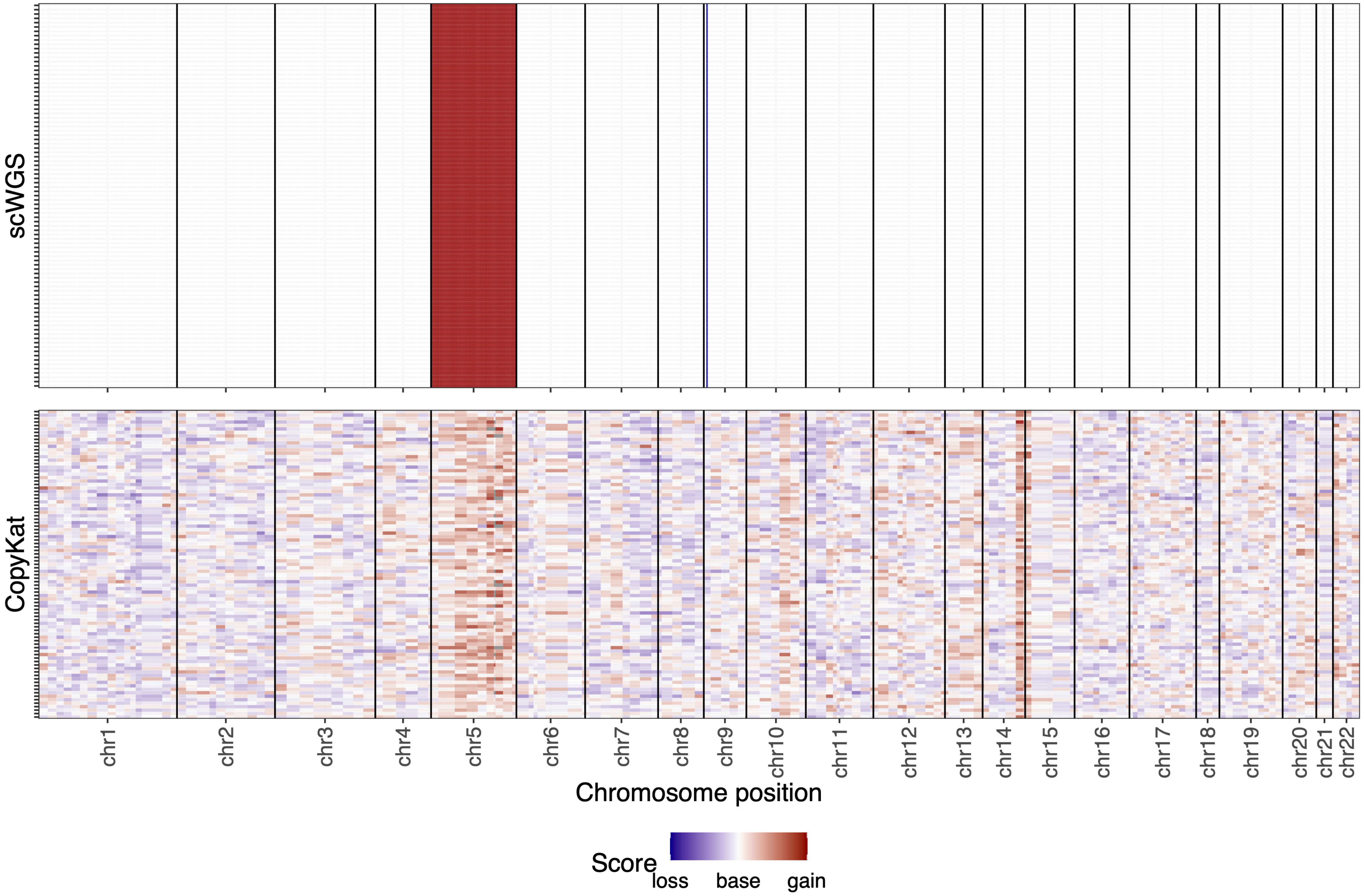

scWGS
CopyKat
chr1
chr2
chr3
chr4
chr5
chr6
chr7
chr8
chr9
chr10
chr11
chr12
chr13
chr14
chr15
chr16
chr17
chr18
chr19
chr20
chr21
chr22
Chromosome position
Score
loss
base
gain

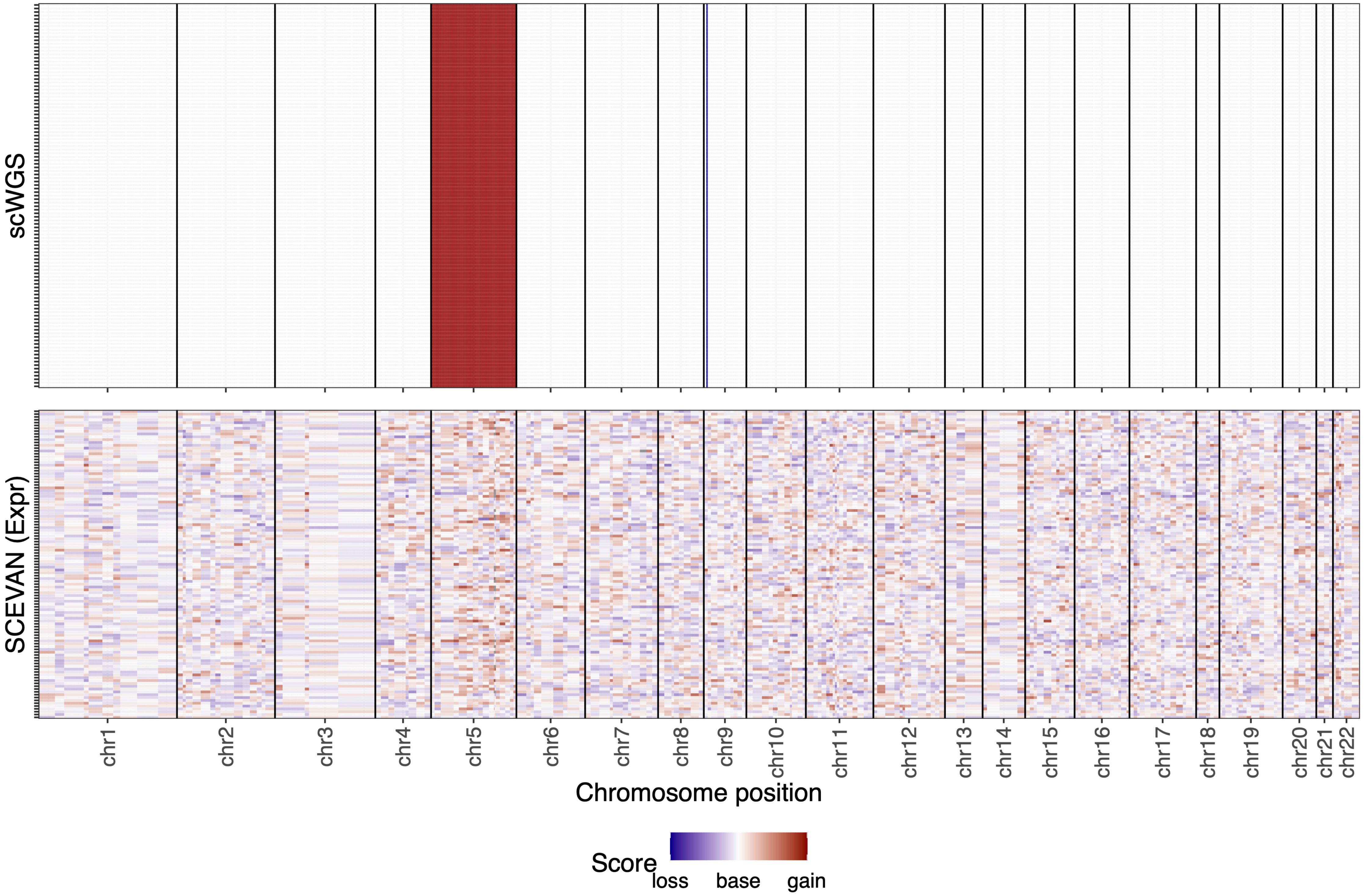
scWGS
SCEVAN (Expr)
chr1
chr2
chr3
chr4
chr5
chr6
chr7
chr8
chr9
chr10
chr11
chr12
chr13
chr14
chr15
chr16
chr17
chr18
chr19
chr20
chr21
chr22
Chromosome position
Score
loss
base
gain

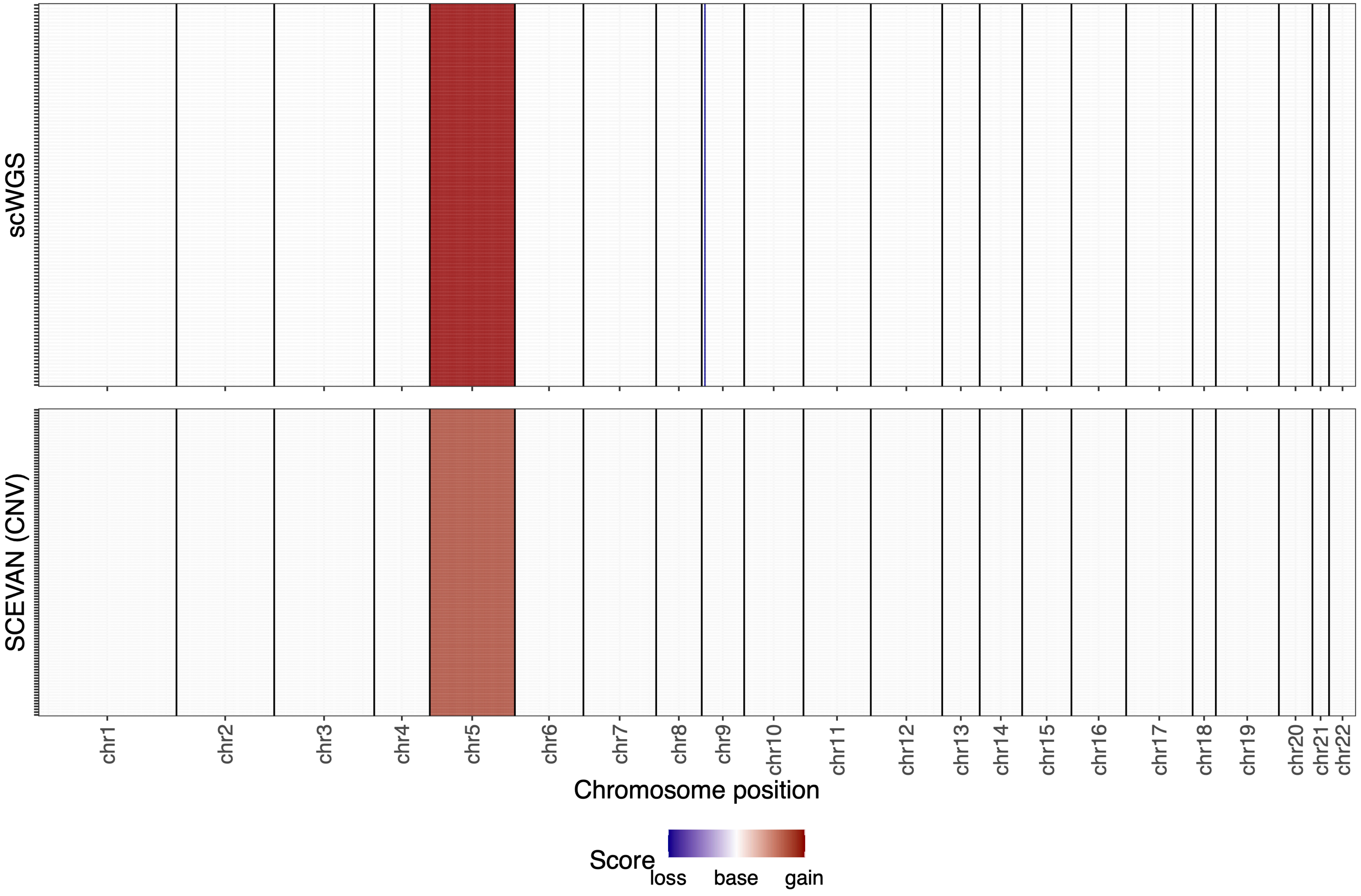
scWGS
SCEVAN (CNV)
chr1
chr2
chr3
chr4
chr5
chr6
chr7
chr8
chr9
chr10
chr11
chr12
chr13
chr14
chr15
chr16
chr17
chr18
chr19
chr20
chr21
chr22
Chromosome position
Score
loss
base
gain

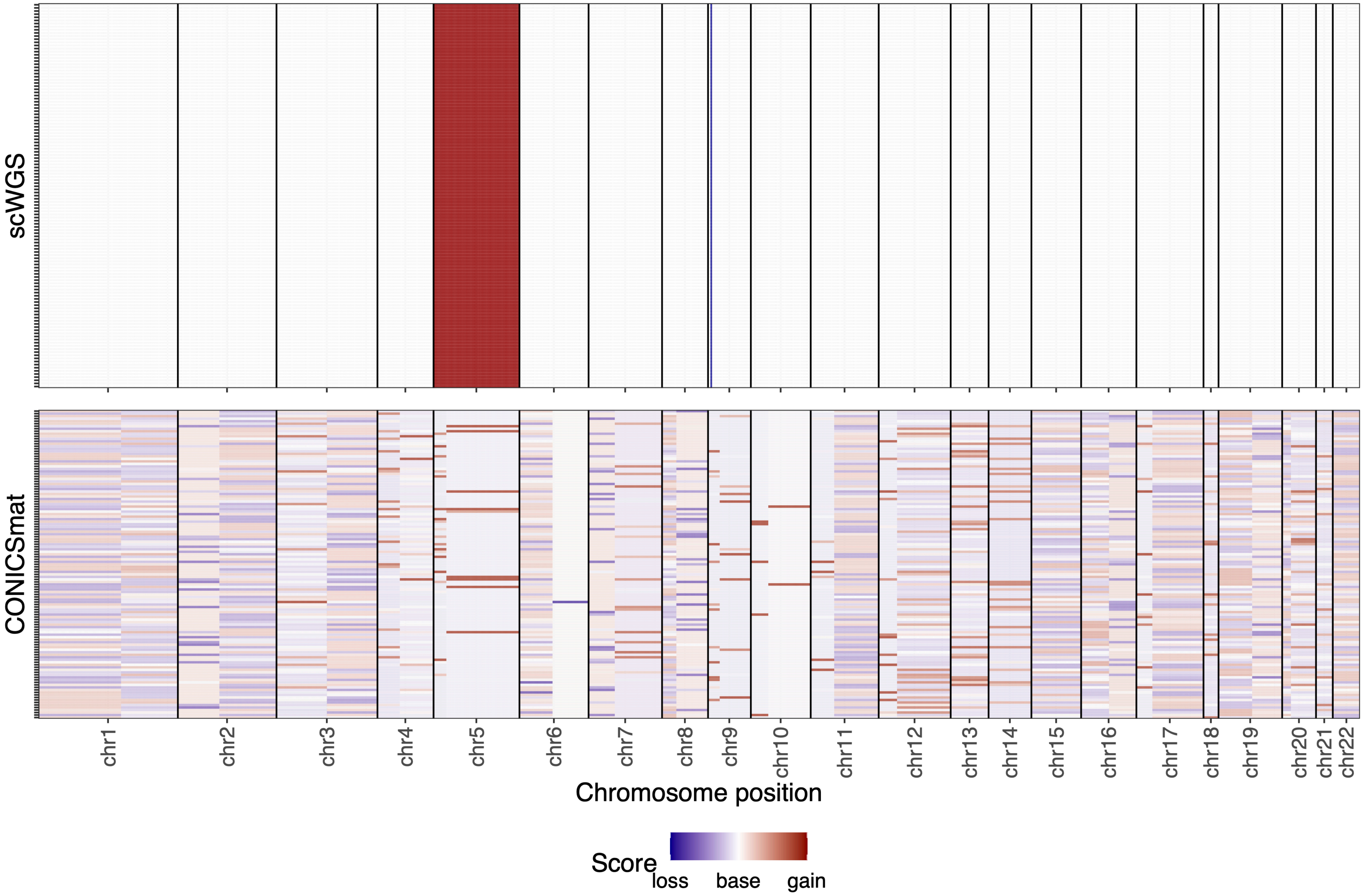
scWGS
CONICSmat
chr1
chr2
chr3
chr4
chr5
chr6
chr7
chr8
chr9
chr10
chr11
chr12
chr13
chr14
chr15
chr16
chr17
chr18
chr19
chr20
chr21
chr22
Chromosome position
Score
loss
base
gain
