# Supplementary material for: Benchmarking scRNA-seq copy number variation callers
Source: Nat Commun. 2025 Oct 2;16:8777. doi: 10.1038/s41467-025-62359-9 (PMC12491403; doi:10.1038/s41467-025-62359-9)
Supplement: Supplementary file 9 — Reporting Summary [file 41467_2025_62359_MOESM9_ESM.pdf]

nature portfolio

Corresponding author(s): Maria Colomé-Tatché

Last updated by author(s): Jun 24, 2025

Reporting Summary

Nature Portfolio wishes to improve the reproducibility of the work that we publish. This form provides structure for consistency and transparency in reporting. For further information on Nature Portfolio policies, see our Editorial Policies and the Editorial Policy Checklist.

Statistics

For all statistical analyses, confirm that the following items are present in the figure legend, table legend, main text, or Methods section.

n/a	Confirmed	
☐	☒	The exact sample size (*n*) for each experimental group/condition, given as a discrete number and unit of measurement
☐	☒	A statement on whether measurements were taken from distinct samples or whether the same sample was measured repeatedly
☐	☒	The statistical test(s) used AND whether they are one- or two-sided<br>*Only common tests should be described solely by name; describe more complex techniques in the Methods section.*
☒	☐	A description of all covariates tested
☐	☒	A description of any assumptions or corrections, such as tests of normality and adjustment for multiple comparisons
☐	☒	A full description of the statistical parameters including central tendency (e.g. means) or other basic estimates (e.g. regression coefficient) AND variation (e.g. standard deviation) or associated estimates of uncertainty (e.g. confidence intervals)
☐	☒	For null hypothesis testing, the test statistic (e.g. *F*, *t*, *r*) with confidence intervals, effect sizes, degrees of freedom and *P* value noted<br>*Give P values as exact values whenever suitable.*
☒	☐	For Bayesian analysis, information on the choice of priors and Markov chain Monte Carlo settings
☒	☐	For hierarchical and complex designs, identification of the appropriate level for tests and full reporting of outcomes
☐	☒	Estimates of effect sizes (e.g. Cohen's *d*, Pearson's *r*), indicating how they were calculated

Our web collection on statistics for biologists contains articles on many of the points above.

Software and code

Policy information about availability of computer code

Data collection	All human data used were publicly available and downloaded from the respective databases (SRA, GEO,ENA). No software was used for data collection. The mouse dataset was generated by co-authors.
Data analysis	All code was deposited on github at https://github.com/colomemaria/benchmark_scrnaseq_cnv_callers . It includes a description of all dependencies as a yml file. We used R 4.1.0 and snakemake (v7.32.4) and tested the following package versions for the RNA CNV callers: inferCNV (v.1.10.0), CONICSmat (v0.0.0.1), CaSpER (v0.2.0), copyKat (v1.1.0), Numbat (v1.4.0), SCEVAN (v1.0.1). The evaluation metrics implemented in R are based on the following R packages: GenomicRanges (v1.46.1), ROCR (v1.0-11), pROC (v1.18.2), crfsuite (v0.4.2). mclust (v6.1.1), clevr (v0.1.2).<br>For mapping of the 10X scRNA-seq data, Cellranger (v7.0.0) was used. For the DNTR-seq data, we used printseq-lite (v0.20.4), trim-gallore (v0.6.11), STAR (v2.7.1a), Picard tools (v3.0.0), HTseq (v2.0.5) and bamtools (v2.5.1). WGS and WES ground truth CNV calls were generated using Trimmomatic (v0.39), BWA (v0.7.17), Picard (v3.0.0), GATK4 (v4.4.0.0), inchorCNA (v0.2.0), CNVkit (v0.9.10), Aneufinder (v1.30.0), bedtools suite (v2.28.0), samtools (v1.8).<br>The mouse data was processed using STAR (v2.7.11a), Bowtie2 (v2.2.4 and v2.3.4.1), BamUtil (v1.0.3) and Samtools (v1.9).

For manuscripts utilizing custom algorithms or software that are central to the research but not yet described in published literature, software must be made available to editors and reviewers. We strongly encourage code deposition in a community repository (e.g. GitHub). See the Nature Portfolio guidelines for submitting code & software for further information.

Data

Policy information about availability of data

All manuscripts must include a data availability statement. This statement should provide the following information, where applicable:

- Accession codes, unique identifiers, or web links for publicly available datasets
- A description of any restrictions on data availability
- For clinical datasets or third party data, please ensure that the statement adheres to our policy

The mouse data generated in this study have been deposited in the Biostudies database under accession code S-BSST1928 [https://www.ebi.ac.uk/biostudies/studies/S-BSST1928].
The previously published datasets used in this study are available here:
For the nine gastric cell lines33, the scWGS data was downloaded from the SRA repository, with accession number PRJNA498809 [https://www.ncbi.nlm.nih.gov/sra/?term=PRJNA498809]. The corresponding scRNA-seq data was downloaded from the SRA repository PRJNA598203 [https://www.ncbi.nlm.nih.gov/sra/?term=PRJNA598203] and the control samples for the scRNA-seq32 data from the GEO repository with accession number GSE150290 [https://www.ncbi.nlm.nih.gov/geo/query/acc.cgi?acc=GSE150290]. A second gastric dataset34 was tested as an alternative reference for the SNU601, downloaded from GEO (accession number GSE159929 [https://www.ncbi.nlm.nih.gov/geo/query/acc.cgi?acc=GSE159929], the stomach dataset GSM4850590 [https://www.ncbi.nlm.nih.gov/geo/query/acc.cgi?acc=GSM4850590]).
For the breast cancer cell line MCF752, ultra low pass whole genome sequencing data was downloaded from the ENA, project PRJNA398960 (Biosample SAMN07519582) [https://www.ncbi.nlm.nih.gov/sra/?term=SAMN07519582], while the scRNA-seq dataset was download for GEO (accession number GSM3142233 [https://www.ncbi.nlm.nih.gov/sra/?term=GSM3142233]). As a reference for the MCF7 cell line, the mammary gland dataset from the Tabula Sapiens cell atlas65 was used [https://figshare.com/articles/dataset/Tabula_Sapiens_release_1_0/14267219].
The multiple myeloma (MM) whole exome sequencing dataset36 was downloaded from the GEO (accession number GSM4200481 for the control sample [https://www.ncbi.nlm.nih.gov/sra/?term=GSM4200481] and GSM4200480 [https://www.ncbi.nlm.nih.gov/sra/?term=GSM4200480] for the tumor sample), while the corresponding scRNA-seq dataset was downloaded from GEO (accession number GSM4200471 [https://www.ncbi.nlm.nih.gov/sra/?term=GSM4200471]).
The COLO320HSR whole genome sequencing dataset58 was downloaded from the SRA accession PRJNA506071 (sample SRS4831935 [https://www.ncbi.nlm.nih.gov/sra/SRX5930165[accn]]), the multiome data66 of the same cell line from the SRA accession PRJNA672109 (sample SRS7587918 [https://www.ncbi.nlm.nih.gov/sra/?term=SRS7587918]), and the control scRNA-seq samples67 were acquired from the gut cell atlas [https://www.gutcellatlas.org/].
The basal cell carcinoma (BCC)35 scRNA-seq samples were downloaded from the GEO repository with number GSE123814 (GSM3511758 [https://www.ncbi.nlm.nih.gov/geo/query/acc.cgi?acc=GSM3511758] for su006 and GSM3511761 [https://www.ncbi.nlm.nih.gov/geo/query/acc.cgi?acc=GSM3511761] for su006 post,GSM3511753 [https://www.ncbi.nlm.nih.gov/geo/query/acc.cgi?acc=GSM3511753] for su005, GSM3511763 [https://www.ncbi.nlm.nih.gov/geo/query/acc.cgi?acc=GSM3511763] for su007 and GSM3511767 [https://www.ncbi.nlm.nih.gov/geo/query/acc.cgi?acc=GSM3511767] for su008). For the BCC sample su006 WES data were downloaded from the SRA accession PRJNA533341 (sample SRS4645189 [https://www.ncbi.nlm.nih.gov/sra/SRX5705755[accn]]).
The two DNTR-seq cell lines29 (HCT116 and A375) were downloaded from the GEO repository, accession number GSE144296 [https://www.ncbi.nlm.nih.gov/geo/query/acc.cgi?acc=GSE144296]. The respective reference cells for the HCT116 cell line68 were taken from the GEO repository with accession number GSE95435 [https://www.ncbi.nlm.nih.gov/geo/query/acc.cgi?acc=GSE95435] and the reference cells for the A375 cell line69 from GEO with accession number GSE151091 [https://www.ncbi.nlm.nih.gov/geo/query/acc.cgi?acc=GSE151091].
The two primary samples of the DNTR-seq data (ALL1 and ALL2) were obtained directly from the authors29. Access to these datasets is restricted, due to being primary patient data, but can be requested from the corresponding author Dr. med. Martin Engee.
The primary sample of ALL with intrachromosomal amplification in chromosome 21 (iAMP21)61 was downloaded from the European Genome-Phenome Archive (EGA). The accession number of the scWGS sample is EGAD00001010288 [https://ega-archive.org/search/EGAD00001010288] and the corresponding scRNA sample is EGAD00001009504 [https://ega-archive.org/search/EGAD00001009504]. The access to these datasets is restricted, being primary patient data. Access can be requested directly through the above links.
The two PBMC datasets were downloaded from 10x Genomics [https://www.10xgenomics.com/datasets/pbmc-from-a-healthy-donor-no-cell-sorting-10-k-1-standard-2-0-0] and GEO accession GSE9658330 (sample GSM2560248, batch A [https://www.ncbi.nlm.nih.gov/geo/query/acc.cgi?acc=GSM2560245]).
Source data are provided with this paper.

Research involving human participants, their data, or biological material

Policy information about studies with human participants or human data. See also policy information about sex, gender (identity/presentation), and sexual orientation and race, ethnicity and racism.

Reporting on sex and gender	All data used for this study were publicly available.
Reporting on race, ethnicity, or other socially relevant groupings	All data used for this study were publicly available.
Population characteristics	All data used for this study were publicly available.
Recruitment	All data used for this study were publicly available.
Ethics oversight	All data used for this study were publicly available.

Note that full information on the approval of the study protocol must also be provided in the manuscript.

Field-specific reporting

[illegible]

Life sciences study design

All studies must disclose on these points even when the disclosure is negative.

Sample size	No sample size selection was made. We chose the datasets to reflect different cancer types, scRNA-seq technologies and organisms as well as a diploid dataset to get a broad representation of use cases for scRNA-seq CNV callers.
Data exclusions	From the SNU601 scWGS dataset a set of 134 cells were excluded from the corresponding comparison, because of their very high ploidy, that did not align with the majority of the cells. Since the cell lines were measured in different laboratories, clonal variation among them can be expected.
Replication	All CNV callers were tested on 14 different cancer datasets to ensure reproducibility of the performance metrics. We provide a snakemake pipeline to ensure reproducibility for other users.
Randomization	No randomization took place in the study. We used all available datasets.
Blinding	No blinding took place in the study. We used all available datasets.

Reporting for specific materials, systems and methods

We require information from authors about some types of materials, experimental systems and methods used in many studies. Here, indicate whether each material, system or method listed is relevant to your study. If you are not sure if a list item applies to your research, read the appropriate section before selecting a response.

Materials & experimental systems

n/a	Involved in the study	
x		Antibodies
x		Eukaryotic cell lines
x		Palaeontology and archaeology
	x	Animals and other organisms
x		Clinical data
x		Dual use research of concern
x		Plants

Methods

n/a	Involved in the study	
x		ChIP-seq
x		Flow cytometry
x		MRI-based neuroimaging

Animals and other research organisms

Policy information about studies involving animals; ARRIVE guidelines recommended for reporting animal research, and Sex and Gender in Research

Laboratory animals	Mus musculus; adult mice, mixed genetic background<br>eT are Msh2-/- mice first published in PMID 7628020 (Cell 1995). T989 are Mad2f/f; p53f/f mice and were first published as tumor model in PMID 28318489 (eLife 2017). The mic were first published in PMID 23382243 (PNAS 2013)
Wild animals	Not applicable
Reporting on sex	In general, to generate CIN+ and CIN- tumours in mice, we used male and female mice and harvested tumours as they arose. In this case we used a tumor from a male mouse (CIN+; T989) and from a female mouse (eT). Sex was not considered in the downstream analyses, as it is not relevant for CNV calling in autosomal chromosomes.
Field-collected samples	Not applicable
Ethics oversight	All animal protocols were approved by the Central Committee for Animal experiments (CCD; permit AVD10500202215846) and UMCG Committee for Animal Care (IvD).

Note that full information on the approval of the study protocol must also be provided in the manuscript.

Plants

Seed stocks	*Report on the source of all seed stocks or other plant material used. If applicable, state the seed stock centre and catalogue number. If plant specimens were collected from the field, describe the collection location, date and sampling procedures.*
Novel plant genotypes	*Describe the methods by which all novel plant genotypes were produced. This includes those generated by transgenic approaches, gene editing, chemical/radiation-based mutagenesis and hybridization. For transgenic lines, describe the transformation method, the number of independent lines analyzed and the generation upon which experiments were performed. For gene-edited lines, describe the editor used, the endogenous sequence targeted for editing, the targeting guide RNA sequence (if applicable) and how the editor was applied.*
Authentication	*Describe any authentication procedures for each seed stock used or novel genotype generated. Describe any experiments used to assess the effect of a mutation and, where applicable, how potential secondary effects (e.g. second site T-DNA insertions, mosiacism, off-target gene editing) were examined.*
